# Spatially Resolved Photostimulation of Cardiac Activity Using Optoelectronic Peptides

**DOI:** 10.1021/acscentsci.6c00713

**Published:** 2026-08-19

**Authors:** Emil M. Lundqvist, Krystal Nguyen, Caleb O. Chung, Sheng Wei Tang, Natalie Celt, Harrison C. Jeong, Sujeung Lim, Lanie Le, Kathryn K. Lee, Kenneth O. Chua, Alessandra Mandala Kol, Jayden R. Stahl, Skylar R. Foust, Yuyao Kuang, Megan N. Jackson, Herdeline Ann M. Ardoña

**Affiliations:** † Department of Biomedical Engineering, 218538 University of California Irvine, Irvine, California 92617, United States; ‡ Department of Chemical and Biomolecular Engineering, 218538 University of California Irvine, Irvine, California 92617, United States; § Department of Chemistry, 218538 University of California Irvine, Irvine, California 92617, United States; ∥ Department of Chemistry, 2331 University of North Carolina at Chapel Hill, Chapel Hill, North Carolina 27599, United States; ⊥ Sue & Bill Gross Stem Cell Research Center, 218538 University of California Irvine, Irvine, California 92617, United States

## Abstract

Engineering physiologically relevant cardiac substrates requires precise control over both electrical and mechanical cues to guide cardiomyocyte functions and features. In this work, we report a biomolecular-based approach toward spatially controllable photostimulation of cardiac behavior via digital light processing of conductive interfaces on a range of substrate stiffnesses. The symmetric peptide-quaterthiophene-peptide units are designed to form conductive patterns atop biopolymeric hydrogels, allowing for microscale spatial control of photoconductive pathways that can influence cardiomyocyte alignment and intercellular communication in a light-activated manner. Substrate stiffness was modulated through adjusting material composition across a physiologically relevant range to reflect healthy and fibrotic cardiac tissue conditions. The ability to tailor the geometry of conductive pathways across a range of biomaterial interfaces and stiffnesses enables systematic assessment of how electrical signals from patterned optoelectronic peptides regulate cardiomyocyte behavior. Neonatal rat ventricular myocytes (NRVMs) cultured on π-conjugated peptide-based substrates exhibited enhanced alignment, synchronous contractility, and altered calcium flux dynamics compared to nonconductive substrates. Furthermore, the optoelectronic cardiac biomaterial-based scaffolds were shown to possess biological photostimulation capabilities, specifically enabling light-induced localized stimulation of excitable cells for high-resolution stimulation without the need for external electrodes or genetic modifications necessary for optogenetic approaches. Photostimulated NRVMs interfaced with optoelectronic peptide structures were shown to be more confined to the printed conductive patterns, with more cell localization and structural remodeling with respect to the patterns than in non-photostimulated conditions. The peptide-based platform presented here provides a potential approach to control mechanical and optoelectronic cues using microscale patterns *in vitro*, while opening avenues for advanced cardiac disease modeling, drug development, and the development of complex bioelectronic cardiac interfaces.

## Graphical abstract

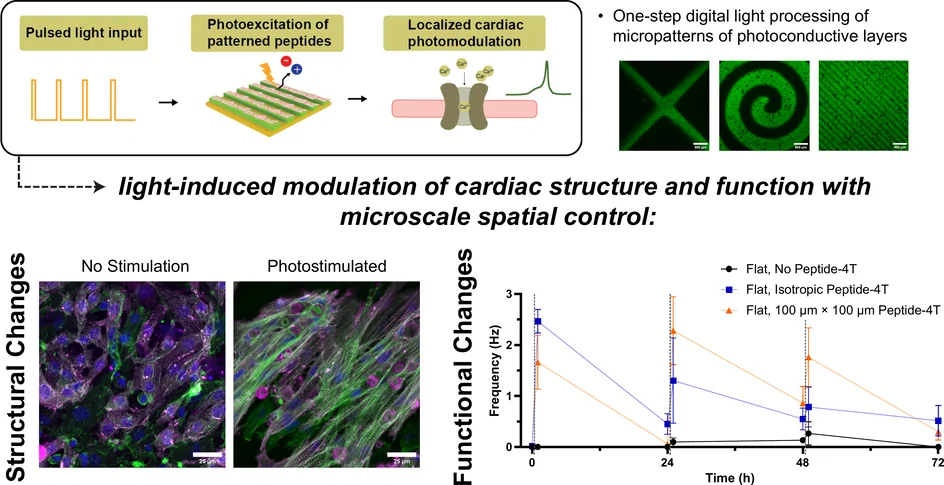

## Introduction

Myocardium is an electrically active tissue where excitable and contractile cells such as cardiomyocytes propagate electrical impulses in a highly coordinated manner to maintain efficient muscle contractions.^1^ The conductive system within the heart comprises electrical impulses initiated at the sinoatrial node, which spread across the atria toward the atrioventricular node and then travel through Purkinje fibers to effectively pump blood throughout the body.^1^ Simultaneously, the myocardial extracellular matrix (ECM) provides a stiffness of ∼5–10 kPa, which influences the sarcomere alignment, calcium handling, and contractile behavior.^2,3^ Calcium (Ca^2+^) signaling triggers cardiac excitation-contraction by releasing Ca^2+^ from the sarcoplasmic reticulum (SR) membrane into the cytoplasm, where the Ca^2+^ binds to troponin and activates the contractile machinery of actin and myosin filaments.^4,5^ Calcium transients rise as calcium moves into cytoplasm during excitation but fall as the calcium is pumped back to the SR membrane and out of the cell during relaxation.^4,5^ When electrical and mechanical features are perturbed, due to myocardial infarction (MI), fibrosis, or deficiencies, damaged tissue is replaced with a collagen-rich fibrotic scar; this disrupts excitation-contraction coupling and results in long-term risk for ventricular dysfunction and heart failure.^1,6^ *In vivo* studies have demonstrated strategies to restore or modulate conduction, including conductive cardiac patches and optogenetic methods in which expression of light-sensitive ion channels enables optical pacing and resynchronization of the heart.^7,8^ While highlighting the importance of conductivity for functional cardiac repair, these approaches are often limited by the lack of precise spatial control over the regions where electric fields are applied and by the inability to independently tune mechanical stiffness within a physiologically relevant range.

To better understand how the dynamic nature of ECM affects myocardial electrophysiology, *in vitro* models have emerged as complementary platforms. These models offer controllable and reproducible environments where conduction, stiffness, and stimulation can be systematically investigated.^9–11^ In particular, a few examples of synthetic materials have demonstrated potential as programmable scaffolds with molecular design that dictates not only biocompatibility and mechanical properties but also dynamic functionality.^10–16^ Such systems enable the incorporation of electronically active motifs, including π-conjugated polymers, graphene/graphene derivatives, or carbon nanotubes, to create conductive networks within otherwise inert matrices that better direct cardiomyocyte electrical signal propagation.^14,15,17–19^ *In vitro* results also demonstrated that performing electrical stimulation on conductive synthetic scaffolds provided efficient electrical communication between cardiomyocytes, thus supporting cell viability and promoting maturation.^20,21^ Yet, the involvement of external electrodes, wires, and rods of conventional electrical stimulation can lead to local damage, contamination at stimulation sites, and importantly, creates a uniform electric field rather than providing high spatiotemporal control over stimulation.^22^ To address the need for more localized stimulation to control field-sensitive tissue functions with spatial resolution, optogenetics-based strategies have been utilized. Unfortunately, this strategy relies on viral transduction to create high-spatiotemporal resolution cardiac stimulation, which can trigger species-dependent immune responses.^23,24^ Incorporating photoconductive functionality into such scaffolds enables light-based stimulation without the need for genetic modifications, which has been shown to successfully accelerate heart-beating rat-based models, thereby opening avenues for wireless and optically controllable cardiac tissues.^25–27^ Especially promising is the potential to codeliver topographical cues with electrical stimuli to introduce a conductive environment while influencing tissue structure. This has been done for anisotropic alignment previously using techniques such as electrospinning^28^ or with combinatorial approaches such as chemical vapor deposition, etching, and deformation;^17^ however, to date, there has been no way to confine the electrical field to localized patterns that can be used to shape photogenerated currents.

To synergistically deliver the structural and conductive cues necessary for guiding cardiomyocyte organization, multiple fabrication strategies have been explored.^10,11,28,29^ Electrospinning produces fibrous scaffolds that showed potential in creating aligned electroconductive fibers that mimic the anisotropic microstructure of myocardium, yet it has limited control of local stiffness heterogeneity and provides bulk electroconductive properties to the cells rather than spatially defined conductive pathways.^28^ To overcome this, we have previously implemented microcontact patterning to deposit photocurrent-generating patterns onto glass (stiff) and gelatin (soft) substrates using water-soluble π-conjugated peptides.^10^ Despite demonstrating that aligned conductive features can direct anisotropic cell organization and alignment, such methods are limited in their ability to control the mechanical microenvironment experienced by cells as they can only modulate bulk stiffnesses by altering the underlying substrate before microcontact printing. Here, we discuss the implementation of digital light processing (DLP) bioprinting, which enables the rapid fabrication of conductive bioscaffolds with user-defined geometry, microscale resolution, and tunable stiffness, achieved through precise spatiotemporal control of photocuring processes. By building geometrically defined substrates layer-by-layer, DLP offers the fabrication of complex, physiologically relevant architectures such as microgroove arrays mimicking aligned myocardial fiber orientation while simultaneously enabling the creation of constructs with flexible geometries such as the cantilevers used in muscular thin film (MTF) assays. Additionally, DLP enables the modulation of stiffness via the adjustment of cross-linking density of hydrogel, grayscale of the photomask, or printing parameters such as ultraviolet (UV) light intensity and printhead speed, recapitulating the mechanical heterogeneity observed in healthy and diseased myocardium.^30,31^ Another advantage of DLP is the ability to create multilayered structures that combine active (stimuli-responsive) and passive layers to achieve selective light-stimulated actuation based on the embedded chromophore and irradiation wavelength.^32^ In our study, we seek to apply a multilayer DLP approach to cardiac scaffolds. By embedding optoelectronic peptides in defined scaffold regions, we can stimulate specific regions of scaffold through light exposure, creating localized conductive pathways and mechanical responses with greater spatial control and complexity than conventional patterning techniques.

In this work, we developed an optoelectronic cardiac biointerface that allows for presentation of underlying photoconductive pathways and stiffness with spatial control. Specifically, we bioprinted photoconductive π-conjugated peptides onto polymeric substrates of methacrylated gelatin (GelMA) for triggering the alignment of cardiomyocytes and elucidating the impacts of biomolecular-based and photoactivated delivery of stimulatory cues on cell organization. The π-conjugated peptides designed for this study comprise a p-type semiconductor, quaterthiophene (4T), and two symmetric peptide sequences at both ends that facilitate cell adhesion and UV-photoresponsivity to cross-link with GelMA in the presence of a photoinitiator under 405 nm light. The functional unit, 4T, has been proven to exhibit photocurrent-generating properties and capability for charge transport, allowing the investigation of electrical signaling of cardiomyocytes, while peptide moieties offer control over adhesive epitope presentation and covalent linkages for substrate stability.^10,26^ By exploiting the precision of DLP bioprinting, we successfully tested a wide range of patterns from microgrooves to more intricate patterns containing curved and sharp-edged features. In parallel, integrating photo-cross-linkable polymers into cardiac biointerfaces permits the generation of stiffness variations to approximate both healthy (*E* = ∼10 kPa) and fibrotic (*E* = ∼55 kPa) states^33^ via the adjustment of GelMA concentration or projected light intensities. In incompressible material environments (those with Poisson’s ratio of ∼0.5, as is commonly assumed for biomacromolecules), the Young’s modulus (*E*) can be approximated as three times the storage modulus (*G′*). As such, we can target storage moduli of ∼3 kPa and ∼18 kPa for the healthy and fibrotic states, respectively. The developed DLP-based approach supports a diverse library of bioinks in addition to GelMA, such as methacrylated collagen (ColMA) and poly­(ethylene glycol) diacrylate (PEGDA), offering increased material modularity and tunable mechanical properties across a wide stiffness range. We systematically investigated how different conduction pathways and mechanical properties impact the alignment and organization of cultured neonatal rat ventricular myocytes (NRVMs), as well as visualized calcium flux dynamics on localized conductive shapes. Finally, the incorporation of an optoelectronic motif enables photostimulation, allowing us to probe the cell localization and synchronicity of beating. Together, this optoelectronic platform provides a pathway to mechanistically dissect how conductivity, stiffness, and light-based stimulation act to regulate cardiac tissue physiology. Through spatially defined photostimulation, and localization of electric fields to direct the current, we demonstrate how cardiomyocytes are impacted both structurally and mechanically by tuning these combinatorial, localized stimuli.

## Results and Discussion

### Design and Fabrication of Optoelectronic Peptide-4T Substrates Via DLP Bioprinting

Organic and hybrid optoelectronic interfaces have previously been studied to enable nongenetic optical stimulation of excitable cells, including conjugated polymer systems that convert pulsed light into interfacial photocurrents capable of modulating membrane contractility.^34–36^ For example, Kuang et al. demonstrated a flexible optoelectronic biohybrid platform based on a poly­(3-hexylthiophene):[6,6]-phenyl C_61_-butyric acid methylester (P3HT:PCBM) donor–acceptor heterojunction and poly­(3,4-ethylenedioxythiophene):poly­(styrenesulfonate) (PEDOT:PSS) carrier-transporting layer, in which light excitation (under 530 nm wavelength) promoted charge-transfer state formation and photocurrent generation at the polymer-electrolyte interface, providing sufficient interfacial stimulation to depolarize excitable cell membranes, trigger action potential generation, and initiate excitation-contraction coupling.^27^ However, these systems rely on preformed multilayer organic semiconductor films rather than water-processable, directly patternable bioinks. Here, the designed optoelectronic peptide-4T integrates a π-conjugated core in supramolecular peptidic moieties, which can be patterned in a single DLP printing step in aqueous conditions. To engineer spatially defined conductive pathways within a physiologically relevant synthetic cardiac matrix, we designed a two-layer DLP bioprinting strategy in which a hydrogel base is first printed and subsequently overlaid with patterned π-conjugated peptide-4T assemblies (Figure 1A). DLP is a photopolymerization-based additive manufacturing technique in which a digital micromirror device (DMD) projects user-defined 2D light patterns onto a photo-cross-linkable bioink, selectively polymerizing entire layers simultaneously.^37,38^ GelMA was selected as the first primary base due to its tunable mechanical properties and high biocompatibility with cardiomyocytes that can mimic the native tissue environment.^37,39,40^ Specifically, GelMA undergoes free-radical polymerization in the presence of the photoinitiator lithium phenyl-2,4,6-trimethylbenzoylphosphinate (LAP) at 0.5 wt % and 405 nm light, covalently cross-linking the naturally derived polymer gelatin and methacrylate groups to form a 3D construct. More importantly, GelMA has been extensively validated as a substrate for cardiomyocyte culture that drives cell attachment and spreading.^41–43^

**Figure 1:**
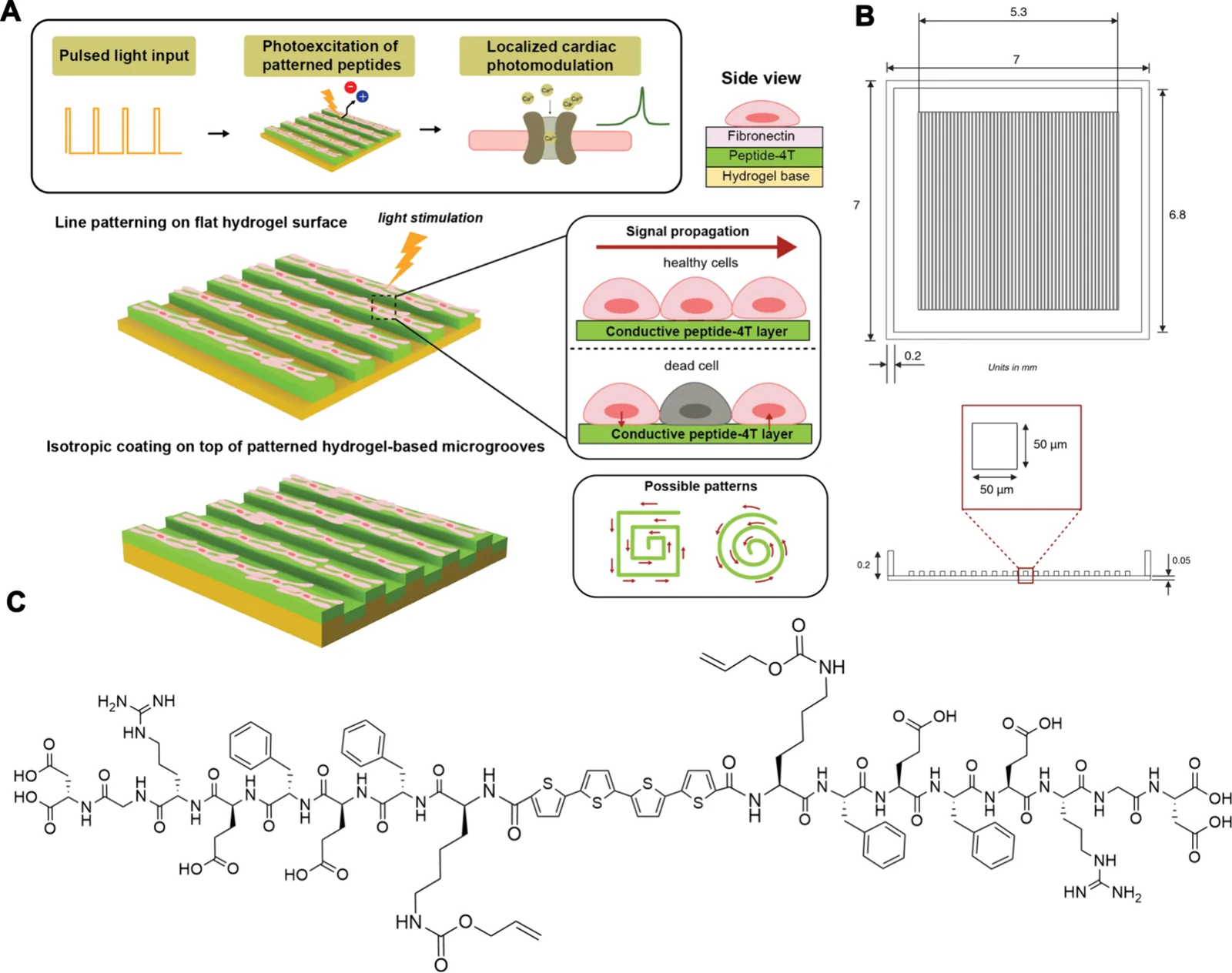
Direct DLP printing of peptide-based photoconductive interfaces. (A) Schematic detailing some of the patterned conduction pathways fabricated via DLP printing and studied here. (B) Top view (top) and side view (bottom) of microgrooved GelMA constructs that can serve as the base for the peptide-4T patterning. Units are in mm. (C) Molecular structure of the symmetric peptide-quaterthiophene material, DGREFEFK_a_-4T.

The hydrogel base layer was fabricated using DLP to produce either a flat or microgrooved architecture (50 μm line width × 50 μm gaps) enclosed by a 200 μm thick and 100 μm tall surrounding wall, yielding a 7 mm × 7 mm × 0.1 mm construct (Figure 1B). This walled geometry is critical for concentrating the NRVM seeding suspension (320,000 cells in 30 μL) within the construct boundaries, ensuring reproducible cell–substrate contact. The microgrooves themselves serve as topographical cues that can guide cardiomyocyte alignment along a defined axis, recapitulating the anisotropic fiber architecture of native myocardium. In our previous work, micropatterned peptide-4T grooves on gelatin significantly improved the orientation order parameter (OOP) of both F-actin and sarcomeric Z-lines in NRVMs compared to isotropic controls and patterned controls without peptide-4T.^10^

The π-conjugated peptide-quaterthiophene designed for this study, DGREFEFK_a_-4T-K_a_FEFERGD (C-terminated), comprises a central quaterthiophene (4T) core flanked by symmetric peptide sequences (Figure 1C). The quaterthiophene-bearing peptide sequence, which was also previously reported for bulk cardiac photostimulation applications,^26^ was selected with the following design considerations: (i) is C-terminated with a known bioadhesive group, arginine-glycine-aspartic (-RGD), an epitope present in the extracellular matrix proteins such as fibronectin;^44^ (ii) bears ionizable glutamic acid (E) and aromatic phenylalanine (F) residues to assist in solubility and templating the ordered assembly of quaterthiophene units under aqueous conditions; and (iii) carries an allyloxycarbonyl- (alloc) functionalized lysine (K_a_) to allow for cross-linking of the optoelectronic patterns to the base hydrogel. Specifically, K_a_ residues serve as reactive handles for covalent cross-linking with methacrylate groups of GelMA under 405 nm light in the presence of lithium phenyl-2,4,6-trimethylbenzoylphosphinate (LAP) photoinitiator. The difunctionalized symmetric design (peptide-π-peptide triblock) used here is particularly beneficial for optimizing π-core interactions, ultimately determining optoelectronic function. When the 4T core is flanked symmetrically, the bonding network from symmetric peptide moieties enforces a geometry that supports π-orbital overlap and helps with effective charge transport. This molecular design consideration is similar to the requirements found in other charge-transporting or optoelectronic peptides, regardless of whether the electron/proton/ion transport occurs via natural residues or by installing active π-conjugated units.^45–49^ The functionalized peptide segment, DGREFEFK_a_, was synthesized using solid-phase peptide synthesis followed by on-resin coupling with π-conjugated quaterthiophene, similar to previous protocols.^26,50^ Together, this molecular architecture integrates three functional roles into a single supramolecular unit: (i) ability to form into bioelectronic 1D assemblies, (ii) covalent anchoring to the GelMA matrix via photopolymerization, and (iii) optoelectronically active π-electron core toward spatially resolved photomodulation of biological activity.

### Photopatterning of Peptide-4T Microgeometries on GelMA Substrates

To validate the versatility and resolution of the DLP printing approach, an ink formulation with 2 mg mL^–1^ of the symmetric DGREFEFK_a_-4T in UltraPure water and 0.5 wt % LAP was successfully printed onto flat 10% GelMA—prepared in phosphate buffered saline (PBS)—substrates with a panel of geometrically distinct patterns, including cross, circular spiral, square spiral, anisotropic lines (100 μm × 100 μm), and a more intricate design with a variety of geometric features depicting the laboratory logo of the Ardoña Research Group (Figure 2A). For all DLP-printed peptide-4T patterns, the printing parameters were consistent with 65% light intensity and 0.001 mm s^–1^ printing speed at a temperature of 37 °C. The patterned 4T chromophore was imaged using confocal microscopy (excitation at ∼488 nm) to provide a quantitative comparison of the printed patterns against the input digital file. Pattern width was measured for each geometry and compared against the nominal design dimensions indicated by the red dashed reference line (Figure 2B). For the spiral geometries, which have a nominal arm width of 500 μm, the pattern printed at approximately 504 μm for both the circular spiral and square spiral, with both distributions showing tight interquartile ranges of approximately ± 20–40 μm. For finer anisotropic line features, we noticed the asymmetric deviation between line and gap widths of 100 μm × 100 μm, where printed. Lines measured slightly above the nominal 100 μm (∼111 μm) while gaps measured below it (∼70 μm). These printing outcomes can be attributed to known lateral ink bleeding during DLP photopolymerization. When employing DLP techniques, the projected light pattern has a finite point spread function, and scattered or diffracted photons at the edge of an illuminated feature can initiate cross-linking slightly beyond the intended boundary.^51,52^ In this case, ink diffusion of peptide-4T into adjacent unilluminated regions reduces the effective gap width while simultaneously broadening printed lines less than expected, a known resolution limitation of hydrogel-based DLP bioprinting at fine-feature scales.^51,53^ Across all geometries, the measured widths remained within ∼10–20% of the nominal design values, confirming that the DLP process accurately transfers user-defined pattern dimensions into the deposited peptide-4T layer with high fidelity across a wide range of feature sizes from 100 μm fine lines to 500 μm spiral arms (Figure 2B).

**Figure 2:**
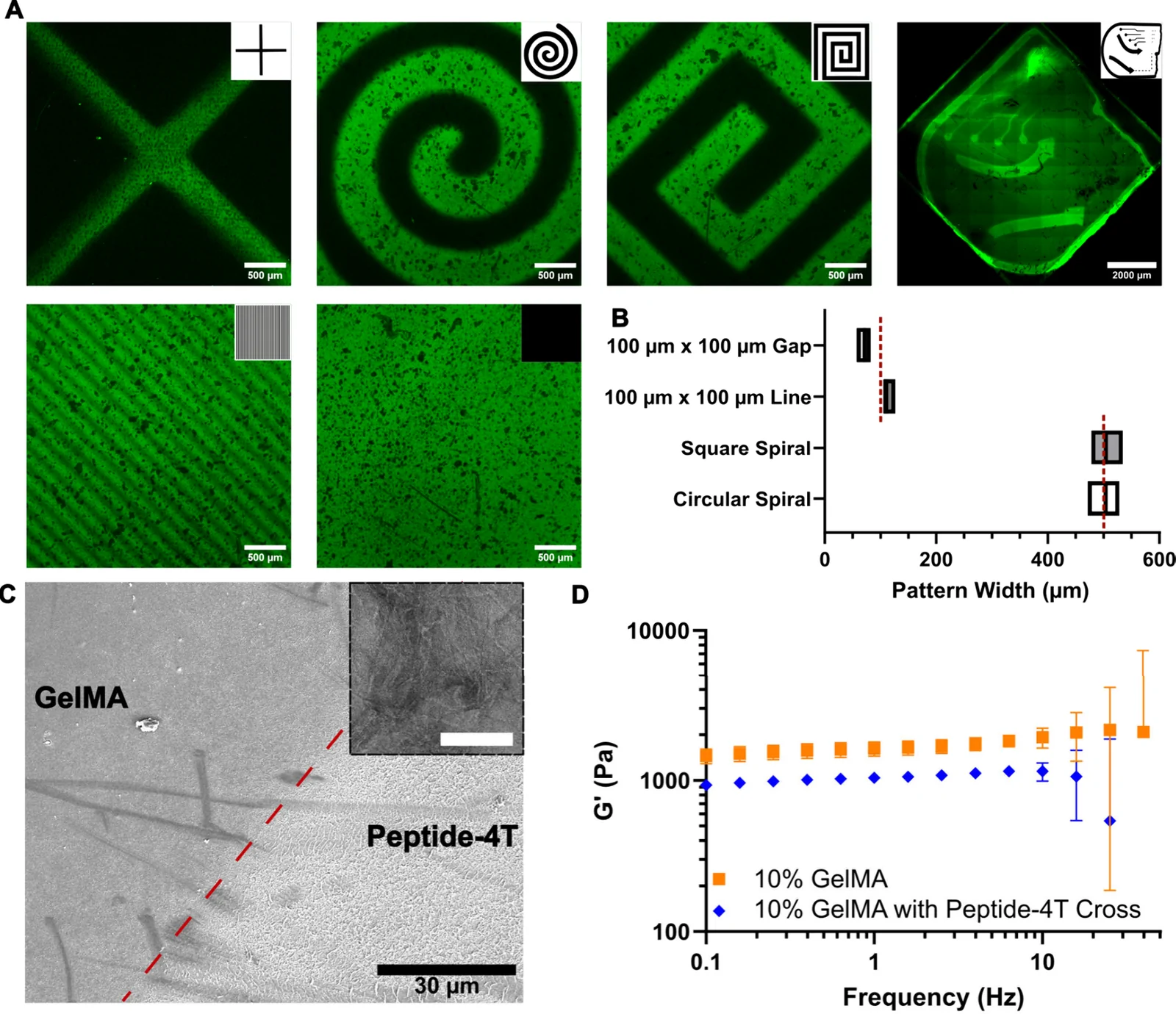
Peptide-4T patterned GelMA substrates. (A) Fluorescence microscopy images of DGREFEFK_a_-4T deposited into various patterns, including a cross, a circular spiral, a square spiral, and a geometrically complex pattern (research group logo). Inset, top right: Input pattern files corresponding to the printed peptide-4T. (B) Measured width of the peptide-4T printed patterns. The actual width from the original input (STL) file is marked by the red dashed line for each pattern to evaluate the relative ratio of deposited peptide-4T to the input parameters. (C) Representative SEM image of a GelMA construct with patterned DGREFEFK_a_-4T deposited on top. The dotted red line indicates the border of the peptide-4T pattern. Inset: representative TEM of drop-cast DGREFEFK_a_-4T showing nanofibrillar structure. Scale bar = 200 nm. (D) Frequency sweeps for 10% GelMA constructs with and without the patterned DGREFEFK_a_-4T on top. *N* = 3 samples per condition. Error bars = standard error of the mean (s.e.m.).

Scanning electron microscopy (SEM) of GelMA constructs with patterned DGREFEFK_a_-4T revealed the surface morphology of the printed structures. The dotted red arrow demarcates the boundary between the DGREFEFK_a_-4T patterned region and the unmodified GelMA surface, confirming that the peptide modifies the surface of the hydrogel, forming a visible compositional boundary of peptide-4T on the GelMA substrate (Figure 2C). The SEM images further reveal fibrillar nanostructures within the patterned region, consistent with the supramolecular self-assembly of DGREFEFK_a_-4T into H-bonding-driven 1D nanostructures (Figure 2C).^10,11,26^ This observation is consistent with the existence of nanostructures on drop-cast films on a glass substrate, visible using transmission electron microscopy (TEM) (inset, Figure 2C; Figure S1). These nanofibers are expected to form a percolating photoexcitable conductive network that supports charge transport across the patterned area, a prerequisite for effective photostimulation of interfaced cardiomyocytes. Using an optical profilometer, the patterned peptide-4T was found to result in an approximate increase of 1 μm in height compared to the surrounding GelMA (Figures S2 and S3).

To assess whether the addition of DGREFEFK_a_-4T layer alters the bulk mechanical properties of the base GelMA hydrogel construct, frequency sweep rheological measurements were performed on 10% GelMA samples with and without DLP-patterned DGREFEFK_a_-4T (Figure 2D). The storage modulus (*G*′) was measured across a frequency range of 0.1–100 Hz at 1% strain. Both conditions exhibited a frequency-independent elastic plateau characteristic of a cross-linked hydrogel network, with 10% GelMA reaching 1800 Pa at 1 Hz. Importantly, the presence of the DGREFEFK_a_-4T pattern did not significantly alter the *G′* of the GelMA base, indicating that the peptide layer integrates with the GelMA surface without disrupting the bulk mechanical integrity of the hydrogel. This mechanical independence is consistent with our prior microcontact printing work, in which peptide-4T overlays on gelatin substrates similarly left the substrate *G*′ unchanged, and confirms that the optoelectronic and mechanical functions of the two-layer constructs can be tuned independently.^10^

### Material Modularity of DLP-Printed DGREFEFK_a_-4T Patterns across Hydrogel Substrates of Varying Composition and Stiffness

Among the design goals of this platform is the ability to independently tune substrate stiffness across a physiologically relevant range while demonstrating that DGREFEFK_a_-4T can be DLP-printed onto chemically distinct hydrogel bases. To this end, three hydrogel systems were evaluated as base materials: GelMA alone (5%, 10%, and 15% w/v), a ColMA:GelMA hybrid, and a GelMA:PEGDA hybrid. The mechanical properties of each hydrogel base were characterized by frequency sweep rheological measurements, showing a frequency-independent elastic plateau characteristic (Figures 3A and S4–S5). The plateau *G*′ values for 5%, 10%, and 15% GelMA ranged from approximately 600–1800 Pa (Figure 3A). While these values are lower than expected given the concentration of GelMA, we believe this lower range in stiffness is the result of the thin (50–100 μm) nature of our constructs. Notably, the addition of a DGREFEFK_a_-4T cross pattern on top of each GelMA concentration did not substantially shift the *G′* values, further confirming that the peptide-4T overlay does not significantly mechanically reinforce or stiffen the hydrogel base. However, the relatively narrow stiffness window of the hydrogels discussed thus far poses the need for further expansion of the mechanical property spectrum for demonstrating both healthy and fibrotic myocardium, motivating the inclusion of hybrid hydrogel systems such as ColMA:GelMA and GelMA:PEGDA. These hybrid hydrogels were printed on constructs of increased thickness (0.5 mm and 1 mm height, respectively) to reduce the potential impact of construct thickness on stiffness. These hybrid constructs resulted in a wider variety of stiffness control, with the ColMA:GelMA reaching an approximate stiffness of 160 Pa at 1 Hz frequency (Figure S4) and stiffness of the GelMA:PEGDA ranging from 11 to 23 kPa depending on the ratio of GelMA to PEGDA (Figure S5). Importantly, none of the bulk stiffnesses of the GelMA:PEGDA construct variations were affected by the inclusion of peptide-4T patterning or coating. The ColMA:GelMA constructs experience an increase in stiffness to about 1 kPa, but this is likely due to the 10 min exposure time used for the alternative coating strategy for these hybrid constructs. The ColMA:GelMA prints required an alternative methodology for the addition of peptide-4T due to the softness of the initial construct; the probe physically compressed and tore the base construct when printing the second (peptide-4T) layer. Considering the range of mechanical properties accessed with a composite biopolymer formulation, these hybrid materials provide an important avenue for future mechanistic studies of myocardium dynamics.

**Figure 3:**
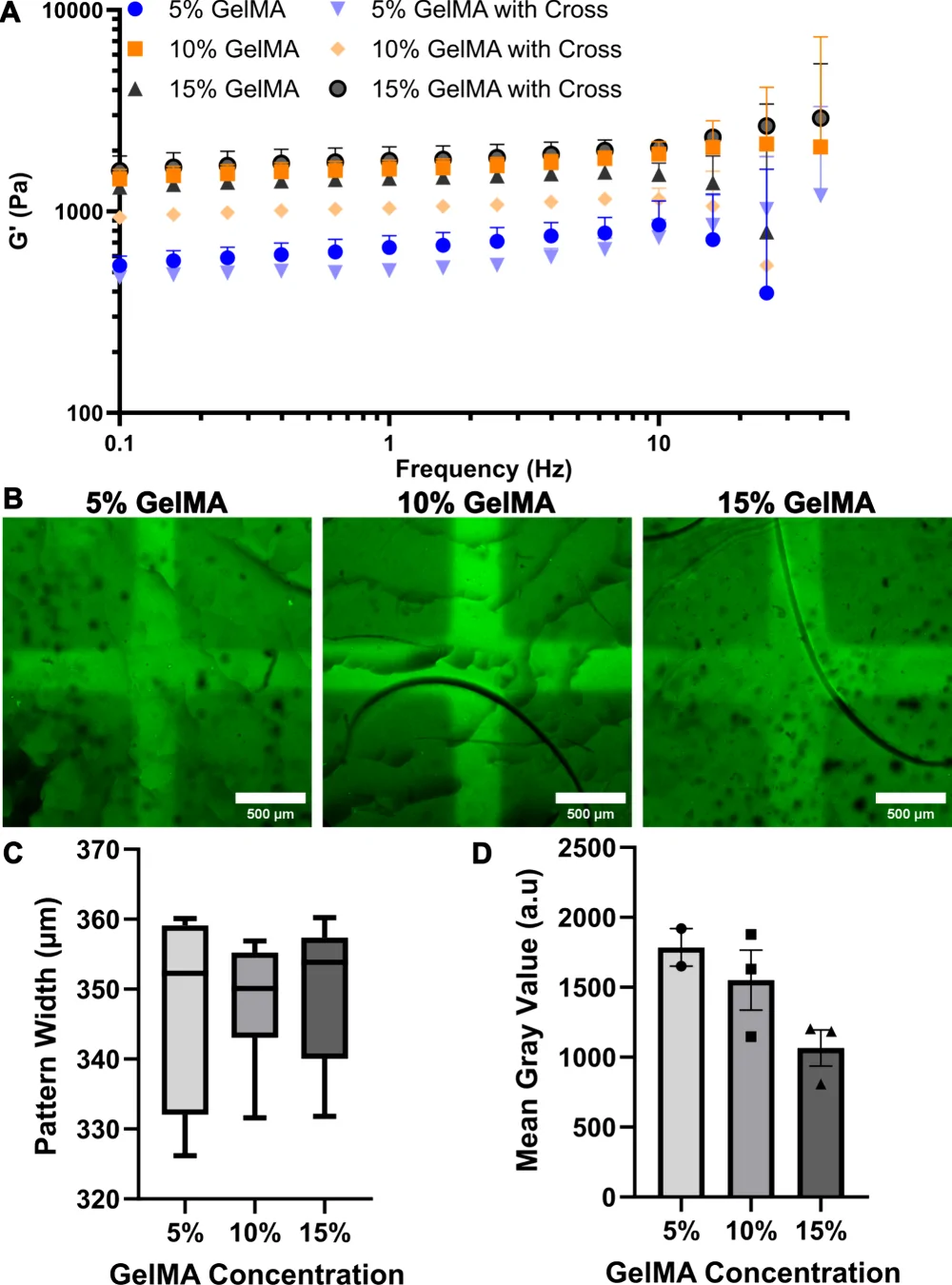
Impact of stiffness on peptide-4T patterning via DLP. (A) Frequency sweep showing the variation in storage moduli resulting from changes in GelMA concentration, with and without the peptide-4T patterning. *N* = 3–4 samples per condition. Error bars = s.e.m. (B) Representative fluorescence microscopy images of 100 μm × 100 μm anisotropic lines printed on the three stiffness substrates, along with (C) the width of the printed DGREFEFK_a_-4T, and (D) the relative fluorescence intensity of the pattern on each stiffness condition. *N* = 2–3 per condition. Error bars = s.e.m.

To assess DGREFEFK_a_-4T patterning fidelity across all three GelMA concentrations, a cross geometry with 400 μm arm width was selected as the test pattern and printed on each substrate. The cross geometry was chosen as a representative test pattern because it contains both line fidelity and feature overlap accuracy. The resulting patterns were characterized by visualization of patterns under fluorescence microscopy (Figure 3B), pattern width measurement (Figure 3C), and mean fluorescence intensity (Figure 3D). Fluorescence microscopy images revealed that the peptide-4T crosses were successfully deposited across all three stiffness conditions, with the 4T chromophore fluorescence providing clear contrast against the nonfluorescent hydrogel backgrounds in each case. For the softer ColMA:GelMA constructs, while the peptide-4T was still able to be isotropically coated onto the gel, the softness of the gel resulted in the DLP printer compressing and destroying the constructs during the subsequent DLP patterning of the peptide-4T (Figure S4). On alternative DLP printers where the print height can be precisely regulated, this issue can be circumvented, allowing for pattern deposition on the extra soft substrates. The stiff 1:1 GelMA:PEGDA was still able to be patterned with the peptide-4T, though the brightness of the pattern was much diminished, potentially due to the lower availability of free acrylate motifs for peptide-4T cross-linking as the cross-linking density of the base matrix increases with increasing *G′*. This outcome is in accordance with the trend of dampened mean fluorescence seen in each of the 5%, 10%, and 15% GelMA constructs.

Pattern width measurements (Figure 3C) showed that the printed arm widths of the cross patterns were broadly consistent across all three hydrogel systems, with values clustering near the 400 μm nominal design width. Within the GelMA series, a trend toward pattern widths closer to the nominal 400 μm at higher concentrations is consistent with the reduced mesh size of more densely cross-linked hydrogels, which limits lateral diffusion of the peptide ink. Next, mean fluorescence intensity was measured by selecting a 100 μm × 100 μm rectangular region of interest within the patterned area of peptide-4T, specifically avoiding large dark imperfections visible on the GelMA surface. The mean fluorescence intensity of the printed cross patterns showed a more pronounced dependence on hydrogel composition (Figure 3D). There is a downward trend in mean intensity of the peptide-4T pattern as the GelMA concentration increases, with intensity values ranging from 1784, 1551, and 1065 au for 5%, 10%, and 15% GelMA constructs, respectively. In addition to considering the availability of free acrylate groups for cross-linking, these observations suggest that greater peptide-4T loading occurs on softer, more porous substrates where the larger mesh size could permit deeper infiltration and entrapment of the peptide nanofibers. This is further corroborated by the stiffer GelMA:PEGDA hybrid composites that exhibited much dimmer, but still successful, pattern transfer (Figure S3). While it is generally established that scattering effects due to ink composition can impact print resolution^53–55^ and previous research shows that the concentration of GelMA can impact light scattering through the hydrogel,^48^ since the difference in the pattern widths on the 5%, 10%, and 15% GelMA constructs are not statistically significant from one another (Figure 3C), it seems likely that any potential scattering effects on pattern deposition are minimal. Taken together, this peptide-4T patterning strategy could be applied in the future to hydrogel libraries with a wide range of stiffnesses, allowing for prospective opportunities to model diseased cardiac environments while incorporating unique peptide-4T patterns.

### Wavelength-Dependent Photoresponsivity of DGREFEFK_a_-4T

To evaluate the potential of DGREFEFK_a_-4T-patterned hydrogels as a platform to drive wireless, optoelectronic stimulation, we utilized sideband Kelvin probe force microscopy (KPFM) to investigate light-induced changes in voltage (Figure 4). Peptide-4T was dropcast onto one-half of an ITO-coated glass, then allowed to dry overnight. After drying, the half-coated ITO substrates were mounted into the KPFM setup for testing (Figure S6). Atomic force microscopy topography images were collected simultaneously with the surface potential in a single pass (Figure 4A–B). On the areas of the ITO glass without any peptide-4T, there was no increase in surface potential in response to 403 or 638 nm light (Figure S7). However, on the part of the ITO glass with the peptide-4T coating, there is a distinct jump in voltage from approximately 200 mV to 600 mV in response to the 403 nm light (Figure 4B–C). This increase in potential in response to the 403 nm light is not present when using 638 nm light, indicating that the optoelectronic properties of DGREFEFK_a_-4T can be driven selectively by using light within the absorbance of the peptide-4T (Figure 4B–C and Figure S8). Also, the lack of significant voltage changes throughout the constant illumination suggests that photothermal effects do not impact photovoltage performance.

**Figure 4:**
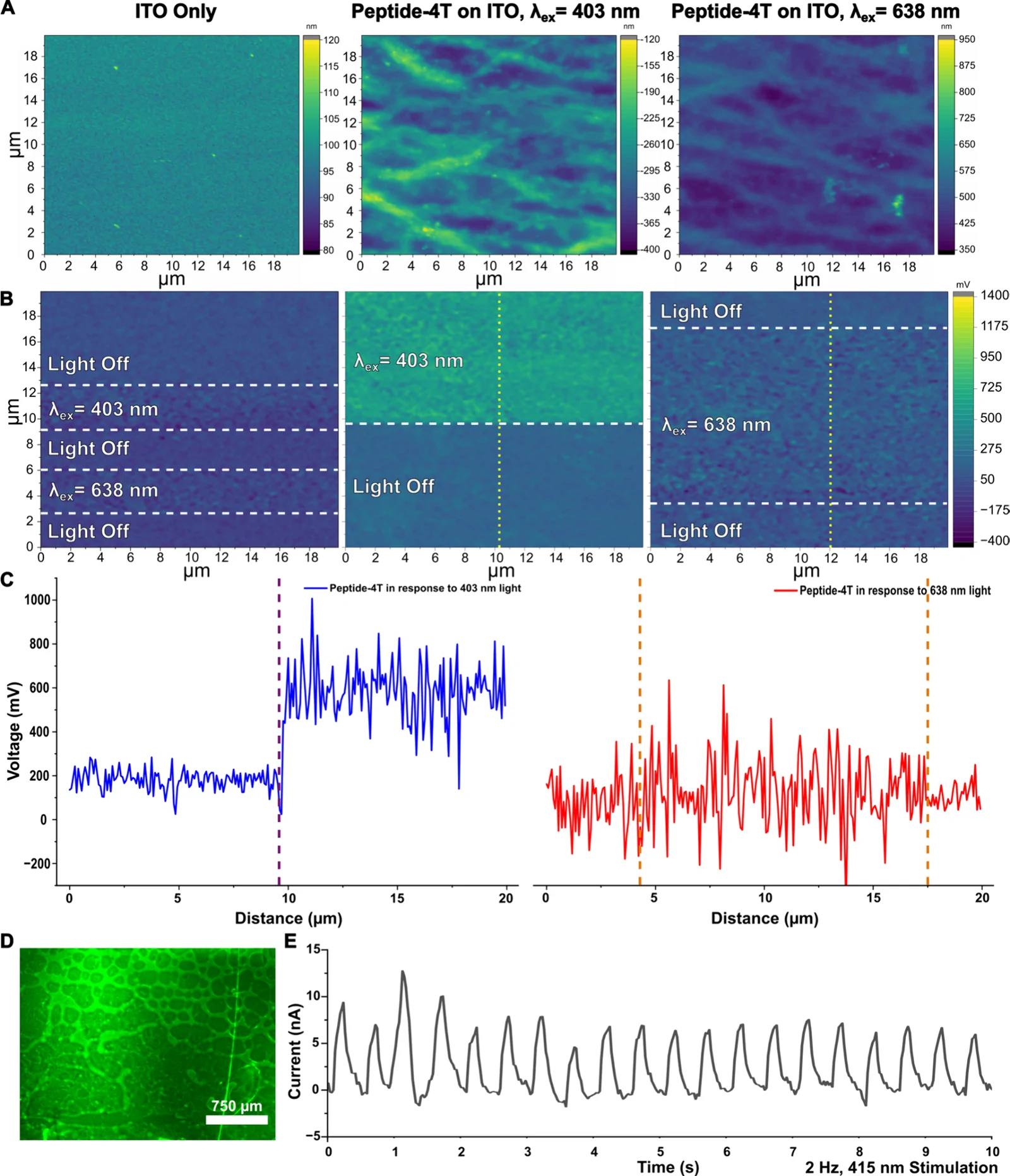
Peptide-4T optoelectronic responses. (A) Atomic force microscopy (AFM) of ITO alone and ITO coated with DGREFEFK_a_-4T taken concurrently with the (B) surface potential measurements of both the ITO and the ITO coated with DGREFEFK_a_-4T during Kelvin probe force microscopy. (C) Graphs depicting the changes in surface potential of DGREFEFK_a_-4T coated ITO in response to 403 and 638 nm light taken along the yellow dotted lines in (B). ITO coated with DGREFEFK_a_-4T shows an increase in surface potential while the 403 nm light is turned on (violet dashed line) but does not have any change in response to the 638 nm light (space between the two orange dashed lines). (D) Fluorescence image of a flat DLP-printed GelMA hydrogel construct cross-linked with 2 mg mL^–1^ DGREFEFK_a_-4T and (E) the current generated from stimulating this sample with 415 nm light at a frequency of 2 Hz.

In order to validate that the photocurrent properties of the DGREFEFK_a_-4T are conserved on the hydrogel system, DLP-printed GelMA substrates were isotropically coated with the peptide-4T and then evaluated for their current generation in response to a 415 nm light pulsed at a frequency of 2 Hz (Figure 4D-E). In response to the 2 Hz stimuli, there is a distinct rise and fall of current synchronous with the 415 nm input stimulus. This signal was not present when probing GelMA alone, where the measurements were erratic and nonresponsive and could not generate a stable measurement.

### Elucidating the Impact of Geometrically Defined Conductive Interfaces on Cardiomyocytes

Having established that DGREFEFK_a_-4T can be DLP-printed with high fidelity across a range of hydrogel substrates and the optoelectronic property of the dropcasted/patterned material, we then investigated whether the resulting bioelectronic interface out of this peptidic material could modulate cardiomyocyte behavior through light-induced photostimulation. NRVMs were seeded onto fibronectin-coated, DGREFEFK_a_-4T-patterned 10% GelMA constructs and subjected to an acute photostimulation regimen of 415 nm light at 2 Hz (100 ms pulse width) at a power density of 27 mW cm^–2^ for 1 h per day on Days 2, 3, and 4 of culture (Figure 5A). This wavelength was selected to match the absorption profile of quaterthiophene chromophore, which generates photocurrent upon illumination and has previously been shown to produce measurable photocurrents of 4.41 ± 0.16 nA under pulsed 415 nm excitation in a related reported DGREFEF-4T system under aqueous electrolytic medium conditions.^26^ Nongenetic, substrate-mediated photostimulation prevents the need for viral transduction or genetic modification of cardiomyocytes, distinguishing it from optogenetic approaches that require expression of exogenous light-sensitive ion channels.^56,57^ Organic semiconductor-based photostimulation platforms have emerged as a promising class of bioelectronic interfaces for cardiac pacing, with prior demonstrations using conjugated polymer films, silicon nanowire matrices, and bioprinted optoelectronic scaffolds to achieve electrode-free,^58^ light-driven cardiac modulation.^25^ However, the materials involved in these previously reported platforms are often not water-processable and therefore not compatible with microscale light-sculpting techniques such as DLP bioprinting with cells for future applications. Furthermore, the light intensities utilized for photostimulation of the DGREFEFK_a_-4T system are significantly lower than those used for inorganic and polymeric semiconductors.^58,59^

**Figure 5:**
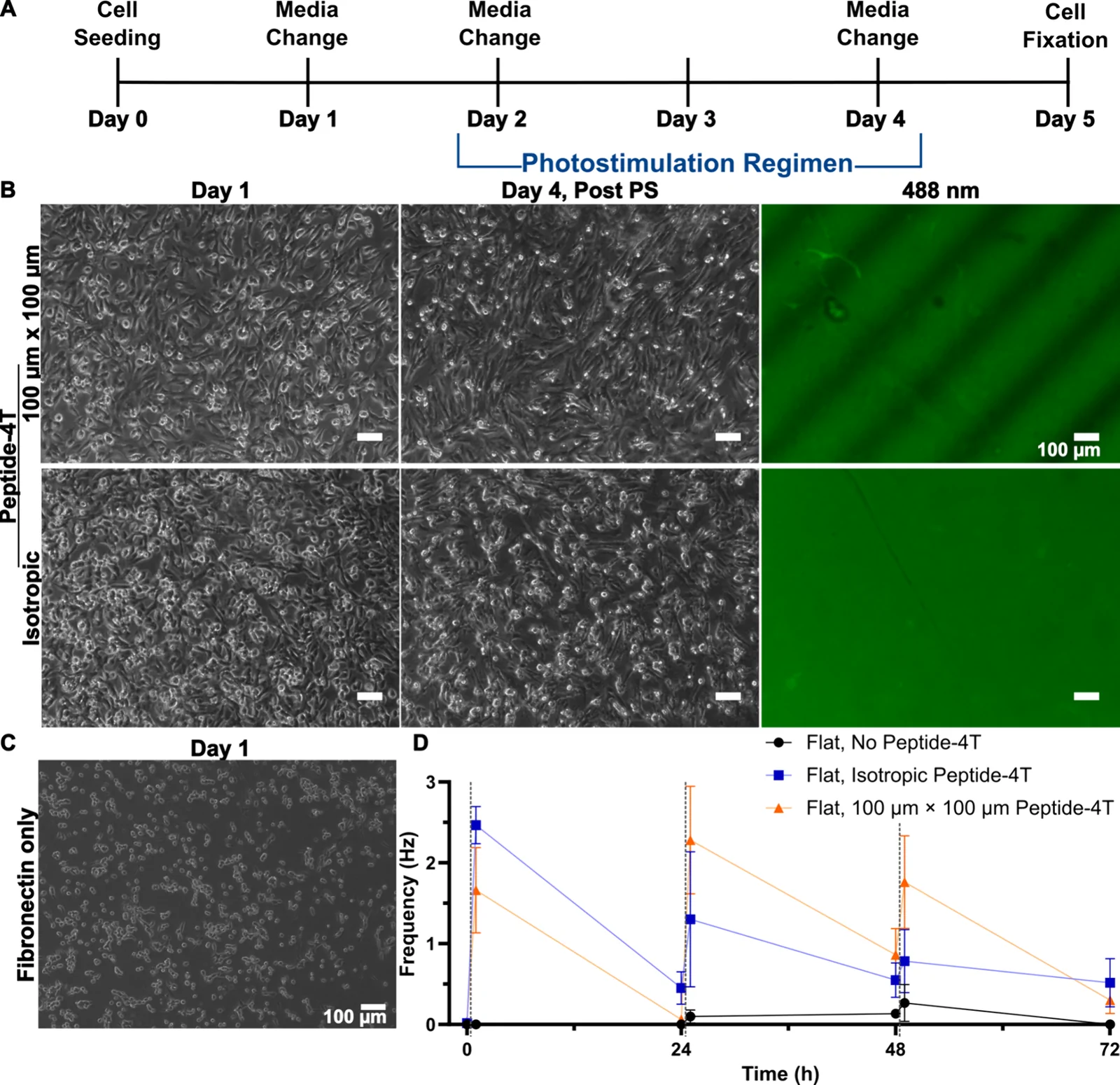
Peptide-4T influence on cardiomyocyte localization and beating. (A) Schematic of photostimulation timelines and cell culture of NRVMs. Photostimulation was conducted using a 415 nm light source on days 2–4 of culture, for 1 h per day at 2 Hz (pulse width duration of 100 ms) at the same regions of interest for each day. (B) Representative images of NRVMs on GelMA constructs coated with fibronectin and a peptide-4T pattern on day 1 and day 4 post seeding after photostimulation (PS). (C) Representative image of NRVMs on a control GelMA construct coated with fibronectin only, without any peptide-4T coating or pattern. (D) Beat frequency of NRVMs on days 2–4 before and after photostimulation and on day 5 before fixation. Photostimulation is marked by the dark gray dashed line. *N* = 5–6 constructs per condition. Error bars = s.e.m.

To investigate how peptide-4T pattern geometry influences cardiomyocyte localization, we tested NRVMs on two DGREFEFK_a_-4T coating configurations on flat 10% GelMA substrates: (i) a 100 μm × 100 μm anisotropic line pattern; and (ii) a uniform isotropic coating, as well as a control 10% GelMA substrate (Figure 5B). These substrates were uniformly coated with fibronectin prior to cell seeding in order to probe the impact of the conductive peptide-4T units in the presence of an isotropically coated bioadhesive unit. Brightfield imaging on Day 1 revealed that NRVMs initially distributed broadly across the construct surface, with cells present both on and off peptide-4T pattern. By Day 4, following 3 days of photostimulation, two conditions diverged markedly. On the 100 μm × 100 μm line-patterned substrate, NRVMs had elongated and adopted a predominantly uniaxial orientation parallel to the underlying peptide-4T line direction, with most cells aligning their long axes along the printed features compared to isotropic substrates. However, cells were not strictly confined to the 100 μm peptide-4T lines themselves but instead spread across both patterned lines and the intervening gaps, forming a continuous but directionally organized cell layer. This is potentially the result of the mismatch between the 100 μm periodicity and the smaller physical dimensions of individual cardiomyocytes. NRVMs on Day 2 of culture have an approximate cell body width of 20–25 μm, meaning that a single cell spans approximately four to five line-gap periods simultaneously.^60^ At this scale, the cell cannot resolve individual 100 μm lines as discrete adhesive boundaries; instead, it integrates the net directional anisotropy of the pattern across its entire footprint, producing alignment of the cytoskeleton and long axis parallel to the line direction without physical restriction to a single line. The ∼50–100 μm practical feature resolution of DLP system used here thus represents a practical limitation for imposing single-cell-scale geometric guidance. Despite the limits in achieving single-cell anisotropy, we demonstrated the ability to systematically vary the pattern geometries to probe how conductive pathway topology could influence cell morphology and organization (Figure S9). Our results also indicated that NRVMs cultured on fibronectin-coated GelMA constructs without any peptide-4T coating showed an isotropic spatial organization. However, without the inclusion of peptide-4T, cells did not preferentially attach to fibronectin-coated GelMA constructs, leading to more sparse constructs (Figure 5C). This further confirmed that the observed localization behavior is attributable to the DGREFEFK_a_-4T pattern rather than the underlying hydrogel microgroove topography or (RGD-containing) fibronectin coating alone.

Beating frequency analysis across Days 2–4 revealed a consistent and reproducible response to photostimulation in both peptide-4T conditions, while no-peptide control remained near 0 Hz throughout (Figure 5D and Figure S10). This demonstrated that baseline beating activity is dependent on the presence of the optoelectronic peptide. Specifically, each photostimulation session (415 nm, 2 Hz, 1 h) produced an acute transient suppression of spontaneous beating immediately poststimulation, followed by a rebound increase at the subsequent prestimulation time point, while the nonstimulated control constructs had a more stagnant beating frequency throughout Days 2 to 5 (Figure S11). Importantly, NRVMs on 100 μm × 100 μm line condition were able to recover to ∼2.25 Hz at 24 h compared to ∼1.3 Hz for the isotropic condition. By Day 5 (72 h after the start of stimulation) without photostimulation, both conditions declined to frequencies ranging from approximately 0.25 to 0.5 Hz, indicating that the beating enhancement is transient and is dependent on the active stimulation period. Additionally, there was not a significant impact on the beating of cardiac tissues when conducting 2 Hz stimulation using 530 nm light, as exemplified by the contractions and beat frequency of the no-peptide controls and the peptide-4T conditions being less than 1 Hz on average (Figures S12 and S13). While the contraction frequency responses of the cardiomyocytes presented in this manuscript do not align 1:1 with the input light stimuli frequency, there is likely a latency between the time scales of the cardiac ionic conduction processes and the optoelectronic generation of the peptide-based material. This latency has been reported for other organic π-conjugated materials used for biointerfacing.^27^ Previous work on polythiophene-based systems and literature indicates that oligothiophenes are primarily dominated by photocapacitive effects; however, we recognize that photofaradaic and photothermal effects may still synergistically play a role in influencing the photoinduced stimulation of cardiomyocytes.^27,34,35^ ^,^ ^61–63^

Viability of cells on GelMA coated with fibronectin only and GelMA coated with fibronectin and an isotropic peptide-4T pattern was evaluated in response to the two different stimulation wavelengths, as well as no stimulation controls, using an MTS colorimetric assay (Figure S14). While there was a significant decrease in the metabolic activity in cells that had been exposed to the 415 nm light compared to both the 530 nm light and no stimulation conditions, cells that underwent the 415 nm light stimulation still showed ∼75% viability. Importantly, there was no significant change in temperature in the wells with the NRVMs on the Peptide-4T coated GelMA before and after stimulation, compared with the no stimulation controls (Figure S15). Furthermore, the peptide-4T pattern was still present after multiple days within the aqueous environment, even with the multiday photostimulation regimen (Figure S16).

### Deconvoluting the Structural Impacts of Topography vs. Optoelectronic Patterns on Cardiomyocytes during Photostimulation

In order to elucidate the potential for using the patterned peptide-4T constructs to introduce photostimulatory signals on cardiomyocytes, we performed a series of experiments intended to deconvolute the organizational influence of passive topographical cues compared to geometrically defined photoconductive patterns. The NRVMs seeded on patterned and isotropic peptide-4T constructs coated with fibronectin, along with 50 μm × 50 μm microgrooved substrates coated with the peptide-4T, were stained for immunofluorescence analysis to evaluate changes in the Z-line (sarcomeric) architecture (Figures 6A-C and S17–S18). Despite all constructs being isotropically coated with fibronectin, photostimulation of the 100 μm × 100 μm patterned peptide-4T resulted in a statistically significant increase in Z-line organization compared to the constructs without the peptide-4T, as shown via the Z-line orientational order parameter (OOP) (Figure 6B). Notably, the photostimulated 100 μm × 100 μm peptide-4T and the peptide-4T coated 50 μm × 50 μm microgrooves saw significant increases in their Z-line organization compared to their nonstimulated counterparts. These results suggest that the underlying microgrooved topography is not necessary for imparting sarcomeric organization, but the effect is only activated once the tissues are subjected to photostimulation. In our previous work, when the line features are closer to the dimensions of single cells, the presence of the patterned optoelectronic peptides alone was sufficient in guiding the cells to organize in an anisotropic manner.^10^ Here, we presented larger features, while additionally subjecting the cells to photostimulation, which showed that the localized fields generated within confined patterns were able to not only transiently impact calcium handling but also enable structural reorganization of sarcomeres with higher anisotropy. Moreover, our previous work did not include the addition of any adhesive matrix such as fibronectin. With the addition of the isotropically coated fibronectin, we see cells localized to the peptide-4T patterns even without photostimulation (Figures S9 and S18). However, the sarcomeres are not organized within these directional optoelectronic patterns unless the photostimulatory regimen is introduced, allowing us to separate the potential impacts of topographical effects and the localized photogenerated stimuli. When the optoelectronic peptides are present, it did not matter whether the topography was printed or if there were physical microgrooves—in both cases, the Z-lines were significantly more aligned and organized. Despite also containing peptide-mediated adhesion, the flat, isotropically coated constructs did not experience a change in the overall Z-line OOP as a result of the photostimulation, even though there were increases in beating frequency during the photostimulation regimen. These outcomes indicate that the patterning of the peptide-4T or the physical patterning of the construct via microgrooves in the presence of peptide-4T, but not the bare microgrooved substrates, can align the force-producing units of the cardiomyocytes. Interestingly, in previous work by Kuang et al. using 25 μm × 4 μm microcontact-printed lines of fibronectin on top of optoelectronic organic photovoltaic blends, NRVMs only show improved alignment of the actin cytoskeleton but not the sarcomeric Z-lines, despite demonstrating the ability to remotely stimulate the cardiomyocytes.^27^ The synergistic impacts of the localized stimuli, peptide-mediated adhesion, and topographical patterning each contribute to the structural remodeling changes of the NRVMs on the light-stimulated, patterned peptide-4T constructs. Additionally, cells seeded on microgrooved samples with only fibronectin and no peptide-4T had consistently poor cell adhesion and confluency, indicating that the optoelectronic units are integral to promoting the adhesion of the cardiomyocytes (Figure S19). Unlike the fibronectin coating, the peptide-4T is directly cross-linked to the GelMA substrates, which may provide more favorable binding sites for the cardiomyocytes and could explain why the discrepancy in NRVM adhesion between the peptide-4T pattern and the control GelMA substrates. Furthermore, each peptide-4T presents two -RGD units at the C-terminus, while each active fibronectin subunit only expresses one -RGD site.^64^

**Figure 6:**
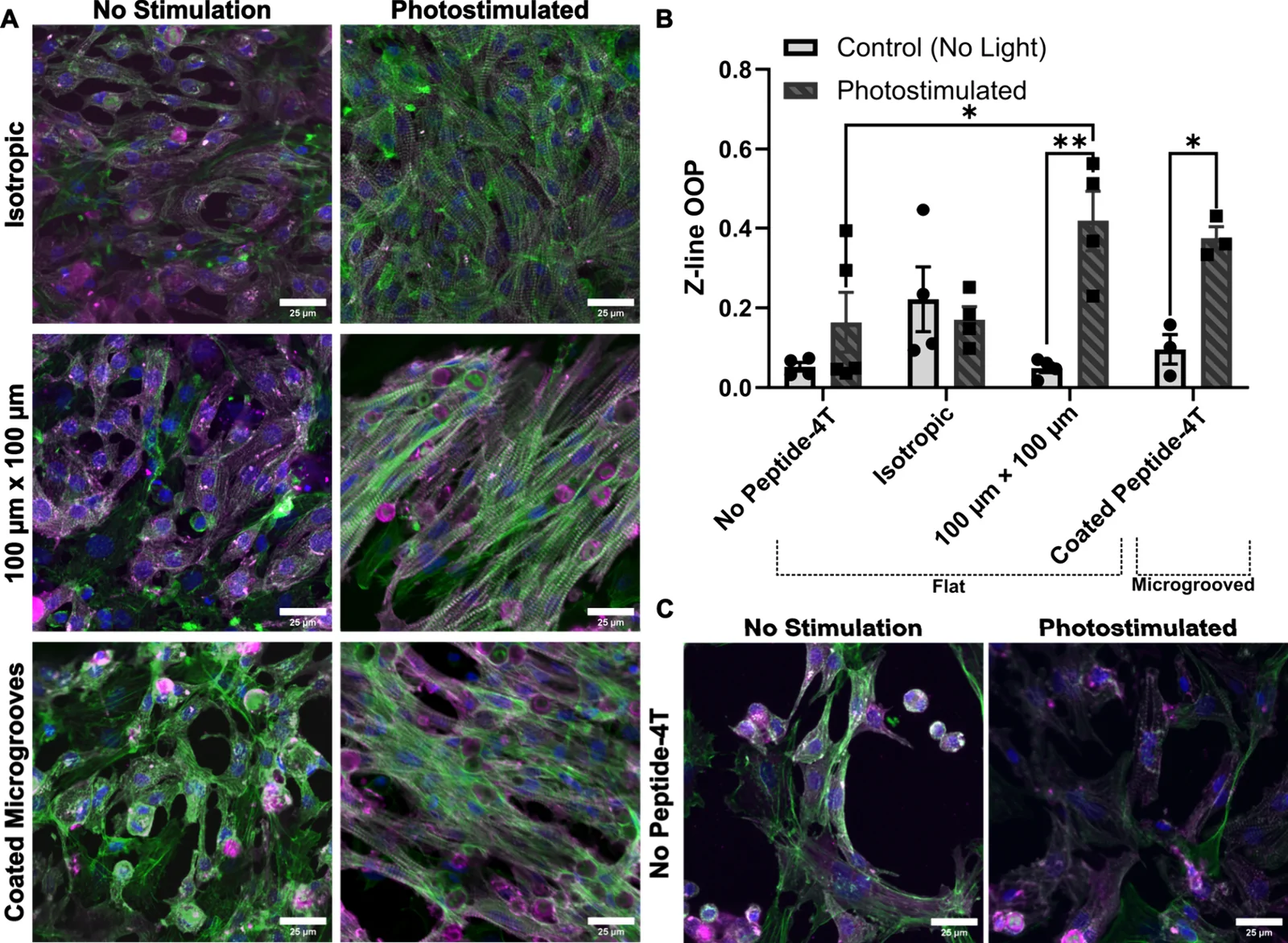
Peptide-4T-induced effects on cardiomyocyte structure and organization. (A) Representative composite fluorescence images of NRVMs seeded on constructs with and without the photostimulation regimen after fixation on day 5. Nuclei, F-actin, and Z-lines are indicated in blue, green, and magenta, respectively. (B) Structural analysis of the OOP of Z-lines for cells on peptide-4T patterns with and without the photostimulation regimen. Statistical significance calculated via one-way ANOVA and Tukey’s HSD. *N* = 3–5 coverslips per condition and 7–13 fields of view per coverslip. *p < 0.05, **p < 0.005. Error bars = s.e.m. (C) Representative images composite fluorescence images of NRVMs seeded on control constructs with no peptide-4T coatings or patterns.

As a proof of concept, the calcium flux of NRVMs seeded on constructs with more unique peptide-4T patterns was studied (Figure S20). In this case, NRVMs were seeded on a flat construct with a circular spiral pattern of peptide-4T, and the calcium flux was visualized using X-Rhod-1, a calcium indicator. Using simple image processing software, such as ImageJ, we can plot and visualize the flow of calcium as it travels along the pattern. We believe that this platform will provide a promising avenue in the future to explore calcium dynamics in environments with various optoelectronic shapes and pathways.

## Conclusions

In this study, we presented the development of a platform that allows for spatial control over photomodulating the biological activity of excitable systems such as in cardiac tissues. To do so, DLP was utilized to create hydrogels that incorporated printable peptide-based optoelectronic microscale patterns. A key advantage of this peptide-4T system is its aqueous processability, which allows the material to be formulated as an aqueous biomolecular-based ink and directly patterned onto hydrogel substrates in a single DLP step. This distinguishes our systems from previous approaches using organic semiconductor systems, which often require sequential film fabrication, conductive interlayers, or nonaqueous processing. The peptide-based molecular design also provides modular tunability, where the peptide domains can be engineered to control the solubility, supramolecular assembly, hydrogel anchoring, and biological ligand presentation. This unique DLP-based patterning technique allows for modular and programmable photoconductive geometries and can be used with a variety of photopolymerizable hydrogel matrices over a wide range of stiffnesses to allow for the modeling of both healthy and diseased tissue states. Cardiomyocytes seeded onto constructs with isotropic coatings of the peptide-4T, as well as patterned peptide-4T, experienced changes in their beat frequency in the presence of light stimulation. Notably, the patterned peptide-4T constructs saw additional changes to the Z-line architecture in response to this photostimulatory signaling as compared to an isotropic presentation of the photoconductive layer, suggesting direct structural effects of geometrically defined photostimulation. A hydrogel-interfaced spatially controlled photostimulation of excitable tissue activity would otherwise not be achievable using conjugated polymers or inorganic materials that are not inherently water-processable. This first-of-a-kind demonstration of microscale-resolved photostimulation via semiconducting peptides therefore offers several advantages for ease of manufacturability for future applications that require upscaling and a higher throughput of platform production. In summary, this work pioneers a modular platform that could advance techniques for controlling cardiovascular signaling dynamics, such as calcium flux, as well as provide a unique approach for introducing light-activated localized stimulatory signals into excitable tissues.

## Materials and Methods

### Peptide-4T Synthesis

Peptide synthesis of DGREFEFK_a_ was performed by solid-phase peptide synthesis (SPPS) using a Liberty Blue microwave synthesizer, as reported in previous work by Yao et al.^65^ The synthetic procedure started with Wang resin preloaded with the first amino acid, Wang-Asp­(OtBu) = 0.6 mmol g^–1^ for DGREFEFK_a_, then sequentially coupled with Fmoc-protected amino acids. Final Fmoc-deprotection was performed by treating resin with 20% piperidine in DMF for 2 min, followed by filtration, and a second treatment with 20% piperidine with another 10 min of mixing. The resin was then sequentially washed 3× with NMP, methanol, and DCM. Following the completion of the oligopeptide sequence and deprotection of the last amino acid residue, the resin was treated with [2,2′:5′,2″:5″,2‴-quaterthiophene]-5,5‴-dicarboxylic acid (0.5 equiv), which was activated by PyAOP (3 equiv) and diisopropylethylamine (10 equiv) for 48 h. Upon completion of coupling, the resin was filtered and washed 3× with NMP, methanol, and DCM. Prior to cleavage, the resin was subjected to another cycle of washes of NMP, methanol, and DCM. The resin was rotated with 9.5 mL of trifluoroacetic acid, 250 μL triisopropylsilane (TIPS), and 250 μL Milli-Q water for 2 h. The peptide solution was filtered from the resin beads, washed 3× with DCM, and was concentrated by a rotovap under reduced pressure and a temperature of 40 °C until the remaining solution was ∼1 mL. The crude peptide started precipitating from solution by adding ∼50 mL of cold diethyl ether and was isolated via centrifugation. The resulting pellet was dried and completely dissolved in water and ammonium hydroxide for dialysis. Crude peptide solution was checked to have neutral pH before lyophilization to obtain the solid product.

### Peptide-4T Characterization

High-performance liquid chromatography (HPLC) was performed to purify the crude peptide products using Agilent 1260 Infinity II Preparative HPLC system (column: Zorbax Eclipse XDB-C8, 21.2 × 250 mm) (Agilent Technologies, CA, USA). The purification was carried out in the mobile phase of 0.1% ammonium formate in Milli-Q water (pH = 8–9) and acetonitrile. Mass spectrum was collected using a Waters Acquity ESI-UPLCQDA (Electrospray Ionization-Ultra performance liquid chromatography-Single Quadrupole Mass Detector) with reversed phase C4 column (Waters Acquity, 50 mm C4) (Waters Corporation, Milford, MA, USA) at the University of California, Irvine Mass Spectrometry facility in negative reflector mode, reported previously in Yao et al. and reconfirmed for this manuscript (Figure S21).^26^ Purity of the samples was confirmed to be 100% pure using analytical HPLC (Zorbax 300SB-C3, 4.6 × 150 mm) (Figure S22). ^1^H NMR spectrum was obtained using a Bruker 2 Avance 500 MHz and processed using MestReNova (Figure S23). Chemical shifts are reported in parts per million (ppm).

Absorption (Figure S8), circular dichroism (CD) (Figure S24), and photoluminescence spectra (Figure S25) were acquired using a Chirascan V100 spectrophotometer (Applied Photophysics, Surrey, UK). Peptides of 1 mM in solution were diluted 10-fold and analyzed immediately by using 1 cm at room temperature. The excitation wavelength for PL is 423 nm, which is the absorption peak of the peptide.

### Printing Via Digital Light Processing

Prior to printing, a 24-well glass-bottom plate was treated with oxygen plasma for 5 min and coated with 10% 3-(trimethoxysilyl)­propyl methacrylate (TMS) in ethanol for 1 h. After removing TMS, each well was washed 5× with ethanol and UV-treated for 30 min. The 3D printing was performed using the BIONOVA X digital light processing (DLP) bioprinter (CELLINK, Gothenburg, Sweden). All patterns were designed on SolidWorks (Dassault Systèmes SOLIDWORKS Corp., Waltham, MA, USA) and sliced using the slicer system of BIONOVA X with each sliced layer being 15–20 μm. The first layer of construct featured a base with central microgrooves of 50 × 50 μm (line × width) surrounded by a wall with 200 μm thickness. The total dimension of the base is 7 × 7 × 0.1 mm (length × width × height). A solution of 10% (w/v) methacrylated gelatin (GelMA) (Advanced Biomatrix, Carlsbad, CA, USA) in 1X PBS solution containing 0.5% (w/v) lithium phenyl-2,4,6-trimethylbenzoylphosphinate photoinitiator (LAP, Advanced Biomatrix) 1X PBS was used for printing the first layer. The printing was performed under 405 nm light at 37 °C, with 85% light intensity, and a printing speed of 0.001 mm s^–1^. Upon the completion of printing, the constructs were washed 3× with sterile PBS to remove residual GelMA. The second layer of the construct involved printing a variation of patterns, including cross, circular spirals, and square spirals, which were designed to fit perfectly into the walls and atop the GelMA-based layer. A solution of filtered 2 mg mL^–1^ peptide-4T in UltraPure distilled water and 0.5% (w/v) LAP was used for printing the second layer. The printing parameters are with 65% light intensity and a printing speed of 0.001 mm s^–1^ at 37 °C. The constructs were washed 3× with sterile PBS to remove residual peptide-4T. Then, the constructs were coated with 50 μg mL^–1^ fibronectin overnight. Peptide-4T patterns were evaluated after printing on either an EVOS M5000 imaging system (Thermo Fisher Scientific Inc., Waltham, MA, USA) or an Olympus Fluoview FV3000 confocal microscope (Olympus, Tokyo, Japan).

### Hybrid GelMA:PEGDA Material Preparation

The hybrid, or composite, DLP-printed constructs were prepared by mixing a 10% weight/volume concentration of methacrylated gelatin (GelMA) containing 0.5% lithium phenyl-2,4,6-trimethylbenzoylphosphinate (LAP) (Advanced Biomatrix, CA, USA) with a 50% PEGDA-INK (700 MW) (Advanced Biomatrix, Carlsbad, CA, USA) containing 0.5% LAP at either a 1:1 or 3:1 ratio (GelMA:PEGDA). All materials were acquired from Advanced Biomatrix. A 1 mm disk of the respective material composition was printed on top of a coverslip to prevent slippage during rheological measurements, using the BIONOVA X using 70% light intensity and 0.05 mm s^–1^ print speed. Hydrogels that were patterned with the peptide were washed with PBS (Thermo Fisher Scientific Inc., Waltham, MA, USA) prior to immersion in the peptide patterning solution and printing with the desired pattern design using the previously described settings. The disk was subsequently washed with PBS three times prior to storing in PBS and conducting rheology.

### Hybrid ColMA:GelMA Material Preparation

Collagen methacrylate (ColMA) solution (Advanced Biomatrix, CA, USA) at a concentration of 3 mg mL^–1^ was acidified (pH 2) to achieve full dissolution. 1000 μL ColMA solution was combined with 700 μL 1 M NaOH and 300 μL HEPES, then the pH was confirmed to be between pH 7–8. The solution was then maintained at 4 °C, to prevent the neutralized ColMA solution from gelling. The hybrid material was designed using the neutralized ColMA solution (3 mg mL^–1^ diluted to 1.5 mg mL^–1^) and 10 wt % GelMA (100 mg mL^–1^). A solution of 1:1 mixture of ColMA and GelMA with LAP concentration at 0.5 wt % was prepared, then kept at 37 °C for DLP printing using the printing parameters discussed earlier. In order to isotropically coat the peptide-4T on the ColMA:GelMA hybrid constructs, the printed constructs were incubated in a 2 mg mL^–1^ solution of peptide-4T for 10 min while exposed to a 415 nm light source.

### Rheological Characterization of 3D-Printed Substrates

The impact of varying stiffness on peptide-based substrates was evaluated using the TA DHR-2 rheometer at the University of California, Irvine TEMPR facility. A parallel-plate cross-hatched geometry (8 mm diameter) was used for the upper geometry, and a standard Peltier plate was used for the lower plate. All experiments were performed at a temperature of 37 °C. The method was adopted from Jeong et al.^66^ Frequency sweeps were performed at 1% strain from 0.1 to 100 Hz with 5 points/decade. The gel was hydrated with water to prevent drying, and DLP-printed hydrogel samples were prepared on adhesive coverslips to prevent slippage and were measured at a constant axial force of 0.1 N (±0.05 N).

### Scanning Electron Microscopy (SEM)

The DGREFEFK_a_-4T constructs were prepared through a sequential dehydration process using an ethanol gradient (10%, 25%, 50%, 75%, and 100%) with 15 min incubations at each concentration. Following dehydration, coverslips underwent critical-point drying (Leica EM CPD 300) (Leica Microsystems, Wetzlar, Germany) and were subsequently sputter-coated (Leica ACE 600) with a 3.0 nm layer of iridium. Imaging was performed at room temperature with a Tescan GAIA3 SEM-FIB instrument (Tescan Group, Brno, Czech Republic) operating at an acceleration voltage of 3.9 kV. All of the equipment used for these experiments is housed within the Irvine Materials Research Institute (IMRI) at the University of California, Irvine.

### Transmission Electron Microscopy (TEM)

A 2 mg mL^–1^ solution of DGREFEFK_a_-4T at neutral pH was prepared with plasma-cleaned 200 mesh copper grids with carbon coating followed by washing and staining with a 1% uranyl acetate solution. The dry samples were imaged by JEOL JEM-2100F (200 kV) (Jeol, Tokyo, Japan) and analyzed by Gatan Digital Micrograph software (Gatan Inc., Pleasanton, CA, USA).

### Optical Profilometry

The surface height of the peptide-4T features on GelMA constructs was measured by using a Keyence VK-X100 3D laser scanning microscope (Keyence, Osaka, Japan). Images were acquired at 10× and 20× magnifications, and 20× images were used for analysis with VK Analyzer software (Keyence). To determine height differences between the peptide-4T-patterned regions and the surrounding GelMA surface, a single line profile (horizontal or vertical) was drawn spanning both regions. The average surface height was calculated within each defined region (peptide-4T and GelMA), and the height difference between the two regions was determined automatically by the software.

### Kelvin Probe Force Microscopy (KPFM)

Sideband Kelvin probe force microscopy images were collected using a Park Systems NX12 (Park Systems, Gwacheon City, South Korea) equipped with an acoustic enclosure, isolation table, and a home-built stage. A piece of double-sided copper tape was placed directly to the top of the half of the sample that was dropcast with DGREFEFK_a_-4T to make electrical contact to the top of the DGREFEFK_a_-4T. Topography and surface potential images were collected concurrently in single-pass mode. PPP-EFM cantilevers (75 kHz) were used. KPFM images were taken in noncontact mode at 0.5 Hz with 256 pixels per line with a free air amplitude of 20 nm and approached to the sample with a set point typically around 11 nm. Driven AC tip bias was 1 V. Sideband frequency was chosen to be ± 3 kHz relative to the resonance frequency of the cantilever. Lock in phase adjustments were set using the sideband autotune function in the SmartScan imaging software. All images were collected with the NX12 instrument light off. An approximately 70 μm by 70 μm square was illuminated underneath the cantilever tip in a ∼150 ms raster scan. Topography images were flattened using the “EZ flatten” function in the Park Systems SmartAnalysis software.

Confocal images of the ITO substrates half-coated with DGREFEFKa-4T were taken prior to KPFM to verify the surface composition of the samples. Confocal images were taken using an extra-long working distance 20×/0.45 objective on a Nikon Ti microscope with an A1 Spectral Confocal detector using NIS-Elements software v6.10.01 (Nikon, Tokyo, Japan). Excitation wavelengths of 403 and 638 nm were used. Images were acquired as 1024 × 1024 pixels.

### Bulk Photocurrent Generation Measurements

A two-electrode setup consisting of tungsten probes was used to measure photocurrent generation of the peptide samples (2 mg mL^–1^) dropcasted onto GelMA. Measurements were made using a Keithley semiconductor parameter analyzer (Tektronix 4200A-SCS-PKB) and pulsing using a Prizmatix 2 Channel LED (415 nm) light source controlled by a Prizmatix pulse train generator (frequency = 2 Hz, pulse width/duration = 150 ms, pulse break = 350 ms).

### Neonatal Rat Ventricular Myocyte (NRVM) Harvest and Culture

Cardiomyocytes were harvested in a similar manner to Lundqvist et al.^10^ Two-day old Sprague–Dawley neonatal rat pups (Charles River, MA, USA) were sacrificed (AUP #23-089), and a portion of their hearts that contained ventricular cardiomyocytes was isolated and then incubated overnight in trypsin (1 mg mL^–1^) (Thermo Fisher Scientific Inc., Waltham, MA, USA), allowing for the dissolution of connective tissue. Following the aforementioned protocol, the ventricular myocytes were purified and then seeded onto the DLP printed constructs. Constructs were designed with a surrounding wall to ensure the NRVMs would be contained within the construct dimensions when utilizing a concentrated seeding system (320,000 NRVMs in 30 μL per construct). NRVMs were then cultured until day 5 post seeding and then processed using the various end point assays (i.e., fixing/immunostaining, calcium flux analysis).

### Calcium Handling

X-Rhod-1 (Thermo Fisher Scientific Inc., Waltham, MA, USA) was prepared at stock concentration of 50 μg μL^–1^ in DMSO, then diluted to optimized concentration of 5 μM using 2% M199. NRVM constructs on day 4 of *in vitro* culturing were incubated with 5 μM X-Rhod-1 for 45 min at 37 °C. After incubation, substrates were washed 3× with PBS and submerged in 2% M199 without phenol red during electrical stimulation and imaging process. NRVMs were recorded with a Zeiss Axio Observer inverted microscope (Carl Zeiss, Oberkochen, Germany).

### NRVMs Immunostaining

Cells were fixed using 4% paraformaldehyde for 10 min, then permeabilized with 0.1% Triton X-100 for 10 min. Cells were incubated with 5% bovine serum albumin (BSA) in PBS for 30 min and then with appropriate primary or secondary antibodies in 1% BSA in PBS for 1 h to fluorescently label actin, nuclei, α-actinin, and vinculin. Substrates were then washed 3× with 0.5% BSA in PBS and 3× PBS. Cells were imaged using an Olympus Fluoview FV3000 confocal microscope (Olympus, Tokyo, Japan).

### Photostimulation

During the *in vitro* culture with NRVMs, 1 h photostimulation at 2 Hz using 415 nm light (power density: 27 mW cm^–2^) was performed on days 2, 3, and 4 to evaluate the behavior of the cardiomyocytes in response to the optoelectronic stimulation (Figure 4A). The beating frequencies of cardiomyocytes were recorded both before and after photostimulation by using a Keyence BZ-X800 epifluorescence microscope (Keyence, Osaka, Japan). Temperature in each well was obtained before and after stimulation by using an infrared thermometer (Etekcity, Anaheim, CA) pointed directly at the construct in the well.

### MTS Metabolic Assay

The MTS metabolic assay was conducted similarly to Lundqvist et al.^10^ Briefly, on day five after cell seeding, the medium in each well was changed to a mixture of 900 μL of Gibco Medium 199 (Thermo Fisher Scientific Inc., Waltham, MA, USA) supplemented with glucose, l-glutamine, vitamin B12, HEPES, MEM-α, and 2% FBS with 100 μL of MTS reagent (Abcam, Cambridge, UK). Cells were allowed to incubate for 0.5 h at 37 °C before 200 μL of media from each well was transferred into a 96-well plate and then analyzed using a Tecan Spark plate reader (Tecan Group Ltd., Männedorf, Switzerland) to measure absorbance at an optical density (OD) of 490 nm. Baseline measurements were conducted at an OD of 650 nm.

### Imaging of Cellular Structures

Fluorescence microscopy images of cells and GelMA-based constructs were taken by using an Olympus Fluoview FV3000 confocal microscope (Olympus, Tokyo, Japan). Fields of view (FOVs) for coverslips were selected by centering the initial field of view to the center of the coverslip, followed by random adjustments in the *x* and *y* axes to select the next field of view in an unbiased manner. Brightfield images of the cells were taken either using an EVOS M5000 imaging system (Thermo Fisher Scientific Inc., Waltham, MA, USA) or a Keyence BZ-X800 epifluorescence microscope (Keyence, Osaka, Japan).

### Structural Analysis of Cardiomyocytes Via MATLAB

The orientational order parameters (OOPs) of Z-lines were calculated in MATLAB (MathWorks, Natick, MA, USA) using analysis codes developed by the Cardiovascular Modeling Laboratory as described in their previous work.^67^

### Statistical Analyses

Statistical analyses conducted for this work were performed via one-way ANOVA, with pairwise comparisons conducted via a *post hoc* Tukey’s HSD test using Prism 9 (GraphPad, San Diego, CA, USA) version 9.5.1.

## Supplementary Material



## Notes

Additional TEM of a dropcast solution of DGREFEFK_a_-4T; fluorescence microscopy images of coated GelMA:ColMA and GelMA:PEGDA hybrid constructs, along with the associated frequency sweeps with storage moduli with and without the peptide-4T; localization of cells on top of peptide-4T patterns with unique shapes, including brightfield images of cells and fluorescent images of the peptide-4T pattern at the same ROI; NRVM beating frequency on peptide-4T constructs and fibronectin only constructs without any photostimulation; immunofluorescently labeled NRVMs on each construct with and without photostimulation; calcium propagation analysis showing frames of a video and the associated regions of calcium spikes; materials characterization for DGREFEFK_a_-4T, including analytical HPLC and ^1^H NMR (PDF)

## ∇

Krystal Nguyen: Department of Chemical and Biomolecular Engineering, Johns Hopkins University, MD 21218, USA
