# Supplementary material for: Spatially Resolved Photostimulation of Cardiac Activity Using Optoelectronic Peptides
Source: ACS Cent Sci. 2026 Aug 19;12(9):1394–409. doi: 10.1021/acscentsci.6c00713 (PMC13614055; doi:10.1021/acscentsci.6c00713)
Supplement: Supplementary file 1 [file oc6c00713_si_001.pdf]

Supporting Information

Spatially resolved photostimulation of cardiac activity using optoelectronic peptides

*Emil M. Lundqvist,[a‡] Krystal Nguyen,[b‡†] Caleb O. Chung,[b] Sheng Wei Tang,[c] Natalie Celt,[a] Harrison C. Jeong,[b] Sujeung Lim,[b] Lanie Le,[a] Kathryn K. Lee,[b] Kenneth O. Chua,[d] Alessandra Mandala Kol,[d] Jayden R. Stahl,[d] Skylar R. Foust,[a] Yuyao Kuang,[b] Megan N. Jackson,[d] and Herdeline Ann M. Ardoña[a,b,c,e]**

[a.] Department of Biomedical Engineering, University of California Irvine, CA 92617, USA.

[b.] Department of Chemical and Biomolecular Engineering, University of California Irvine, CA 92617, USA.

[c.] Department of Chemistry, University of California Irvine, CA 92617, USA.

[d.] Department of Chemistry, University of North Carolina at Chapel Hill, NC 27599, USA.

[e.] Sue & Bill Gross Stem Cell Research Center, University of California Irvine, CA 92617, USA.

‡Denotes equal contribution

*Corresponding Author: hardona@uci.edu

KEYWORDS: photoconductive materials, peptides, biomaterials, cardiac tissue engineering, patterning

Table of Contents

Peptide-4T Transmission Electron Microscopy **3**

Optical Profilometry of Peptide-4T GelMA Constructs **4-5**

Hybrid GelMA Constructs **6-7**

GelMA:ColMA Hybrid 6

GelMA:PEGDA Hybrid 7

KPFM for Peptide-4T on ITO **8-9**

KPFM/AFM Setup 8

Surface Potential of ITO Control 9

DGREFEFK$_a$-4T Absorbance Spectrum **10**

Cell Localization on Peptide-4T Patterns **11**

Beat Rate of NRVMs on Photostimulated DGREFEFKa-4T Constructs **12**

NRVM Beat Frequency without Photostimulation **13**

NRVM Beat Frequency with 530 nm Photostimulation **14**

Beat Rate of NRVMs on 503 nm Photostimulated DGREFEFKa-4T Constructs **15**

MTS Viability Assay for Photostimulated Samples **16**

Well Temperature before and after Photostimulation **17**

DGREFEFK$_a$-4T Pattern Stability **18**

NRVM Immunofluorescence Structural Analysis **19-21**

With Photostimulation Regimen 19

Without Photostimulation 20-21

Calcium Flux Analysis **22**

Additional Materials Characterization 23-27

DGREFEFK$_a$-4T ESI-MS Spectrum 23

DGREFEFK$_a$-4T Analytical HPLC Trace 24

DGREFEFK$_a$-4T ^{1}H-NMR Spectrum 25

DGREFEFK$_a$-4T Circular Dichroism Spectrum 26

DGREFEFK$_a$-4T Photoluminescence Spectrum 26

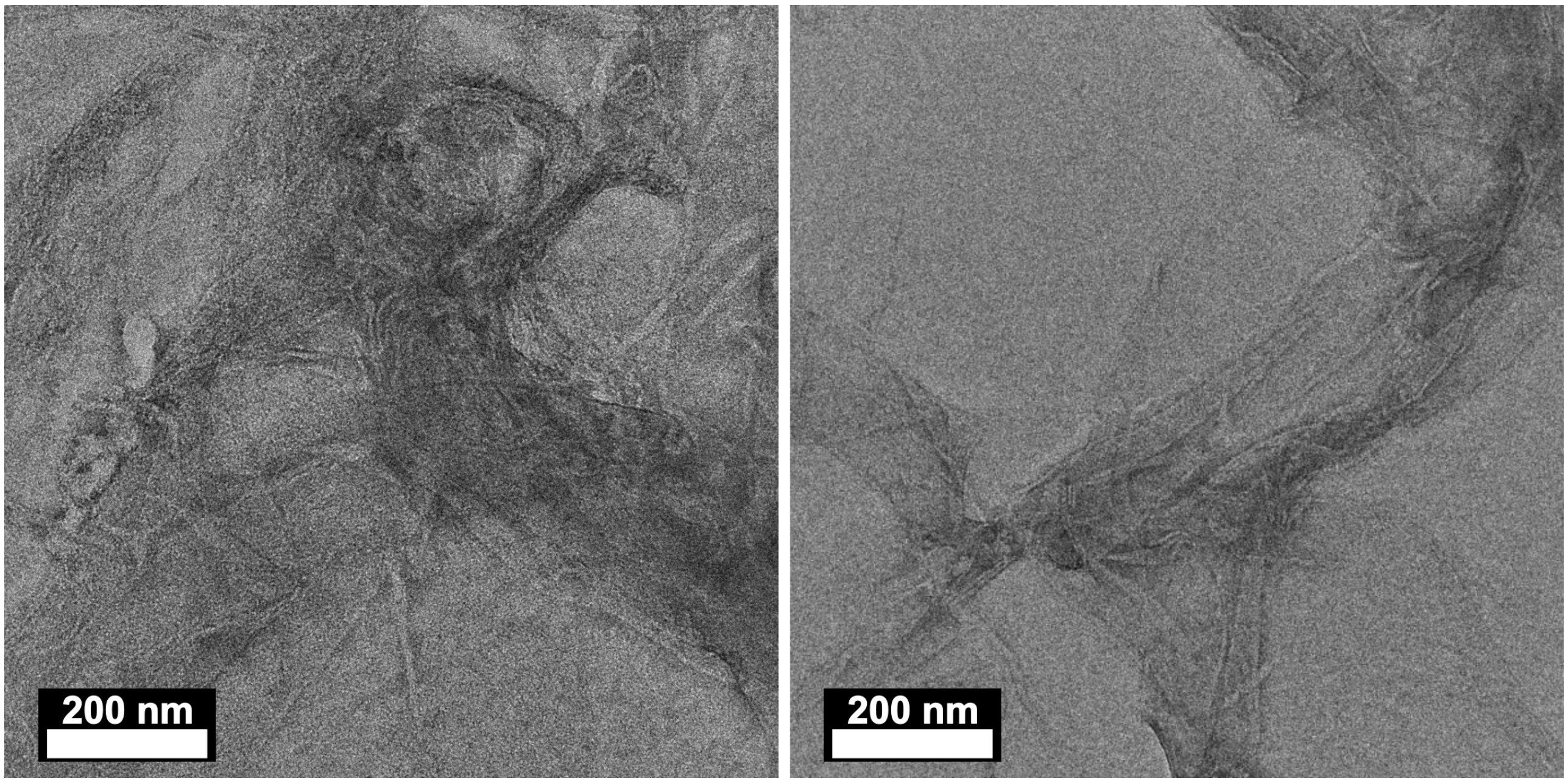

Figure S1. Transmission electron microscopy image of dropcast DGREFEFK$_a$-4T showing nanofibrillar structure.

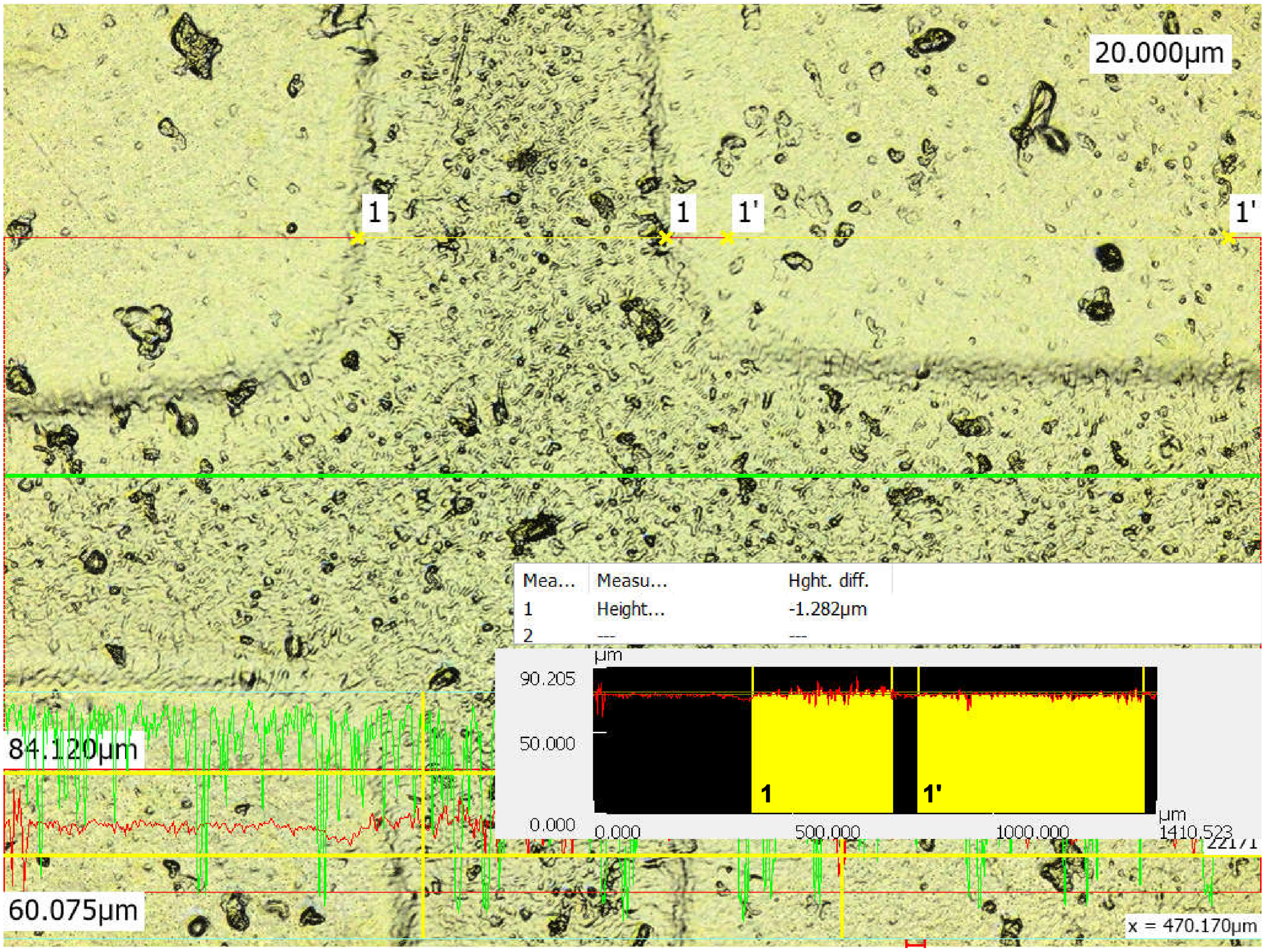

Figure S2. Profilometry images showing height differences between a printed peptide-4T cross pattern (marked by 1) and the surrounding GelMA (marked by 1'). In the selected region, there is a 1.282 µm increase in height at the location of the peptide-4T pattern.

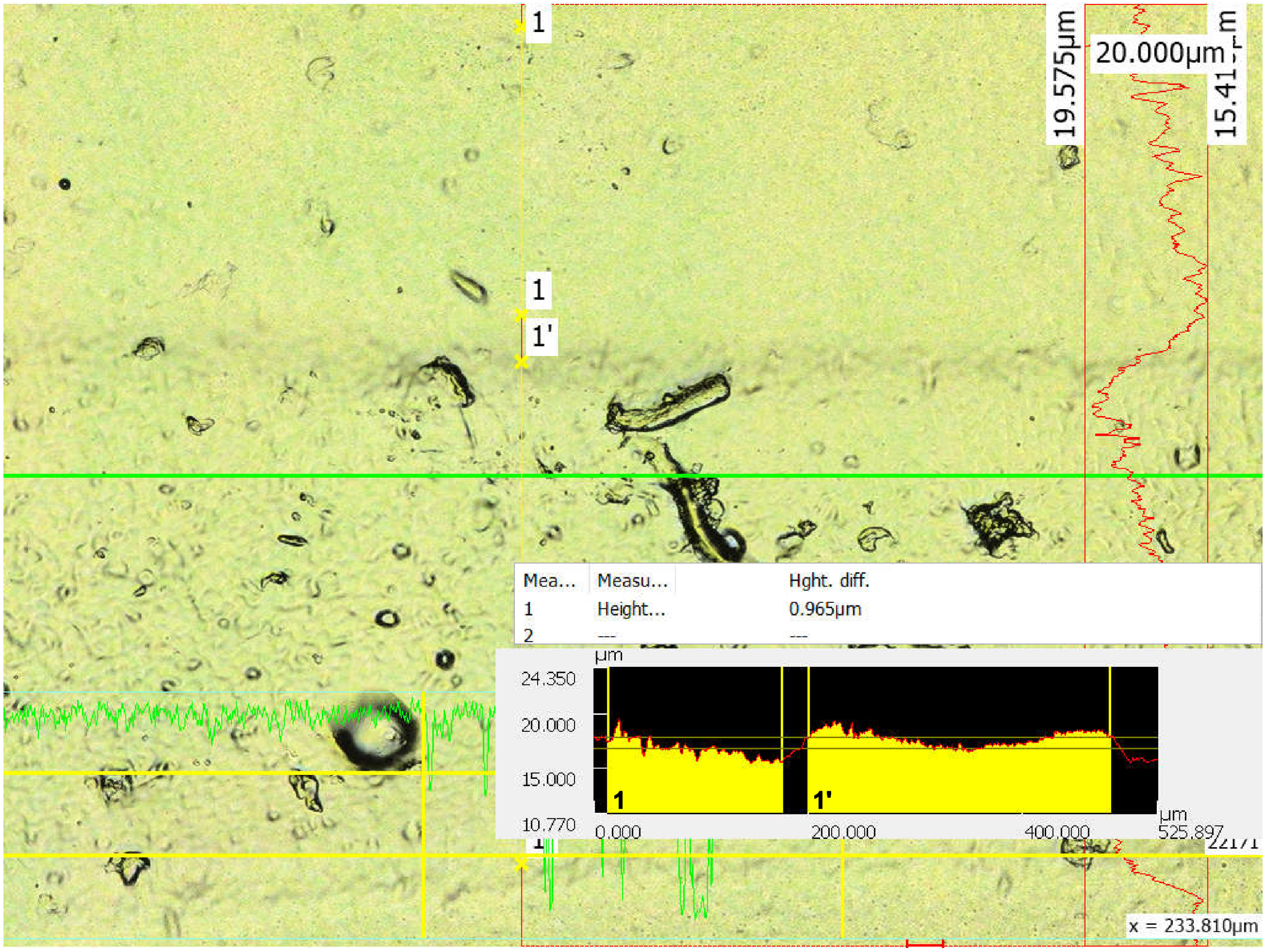

Figure S3. Profilometry images showing height differences between a printed peptide-4T cross pattern (marked by 1') and the surrounding GelMA (marked by 1). In the selected region there is a 0.965 µm increase in height at the location of the peptide-4T pattern.

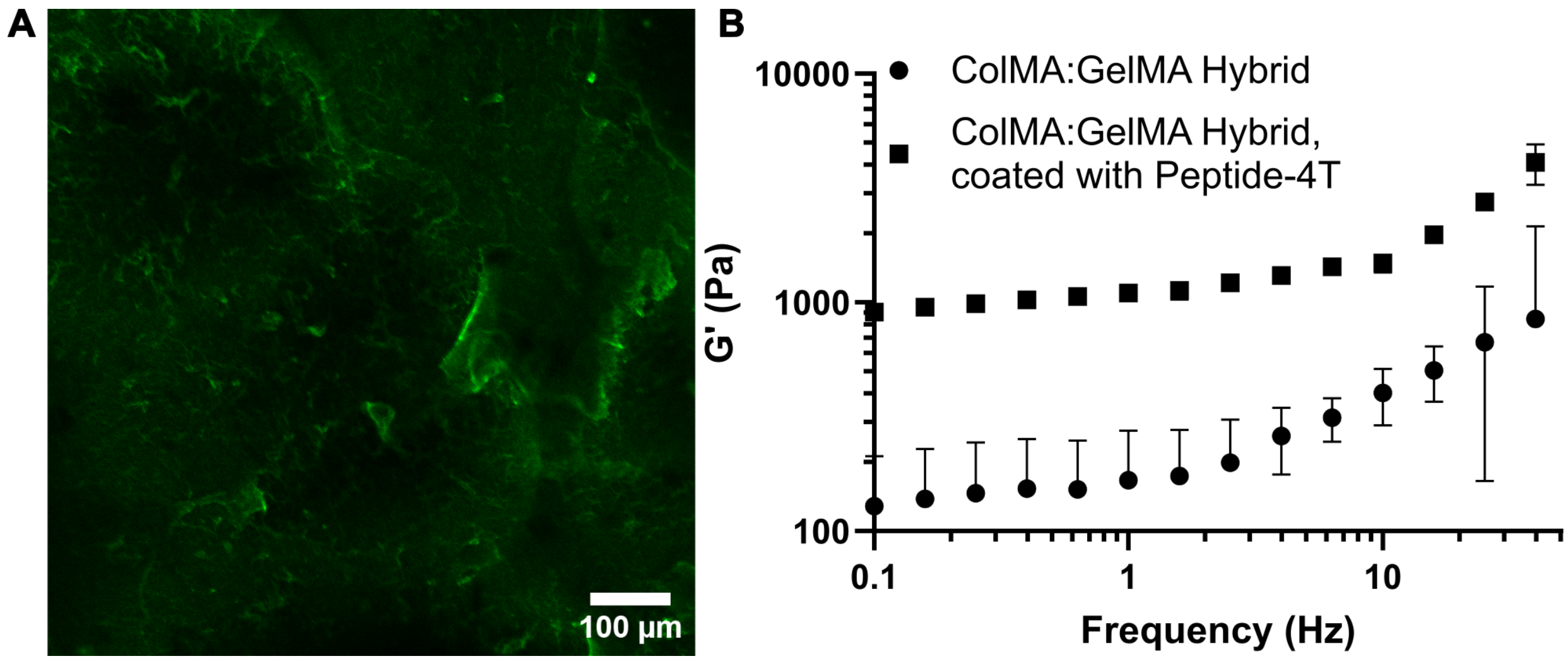

Figure S4. GelMA:ColMA Hybrid constructs. (A) Fluorescent image (contrast adjusted) of the isotropically coated DGREFEFK$_a$-4T on a GelMA:ColMA construct. (B) Frequency Sweep of 1:1 GelMA:ColMA constructs with and without the isotropic coating of DGREFEFK$_a$-4T. N = 3 per condition. Errors bars = s.e.m.

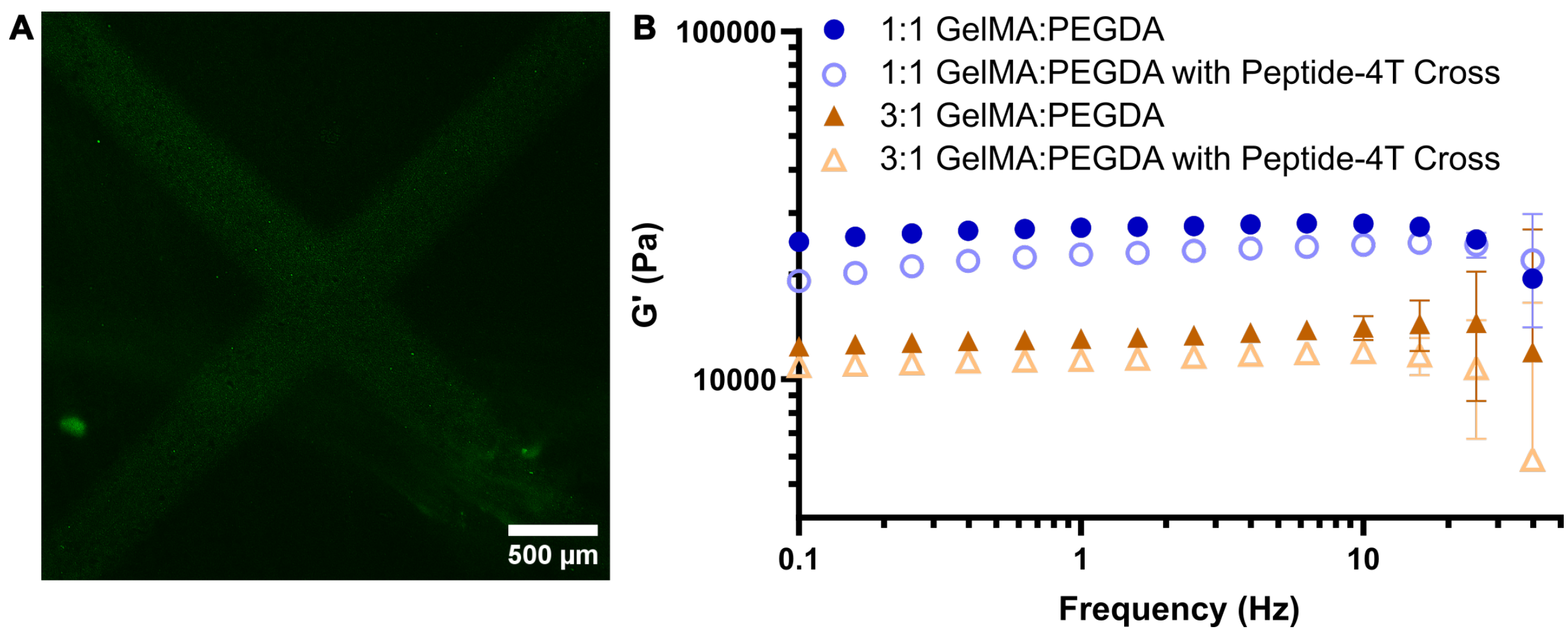

Figure S5. GelMA:PEGDA Hybrid constructs. (A) Fluorescent image (contrast adjusted) of the DGREFEFK$_a$-4T cross pattern printed on the 1:1 GelMA:PEGDA construct. (B) Frequency Sweep of 1:1 and 3:1 GelMA:PEGDA constructs with and without the patterned peptide-4T printed on top. N = 3 per condition. Errors bars = s.e.m.

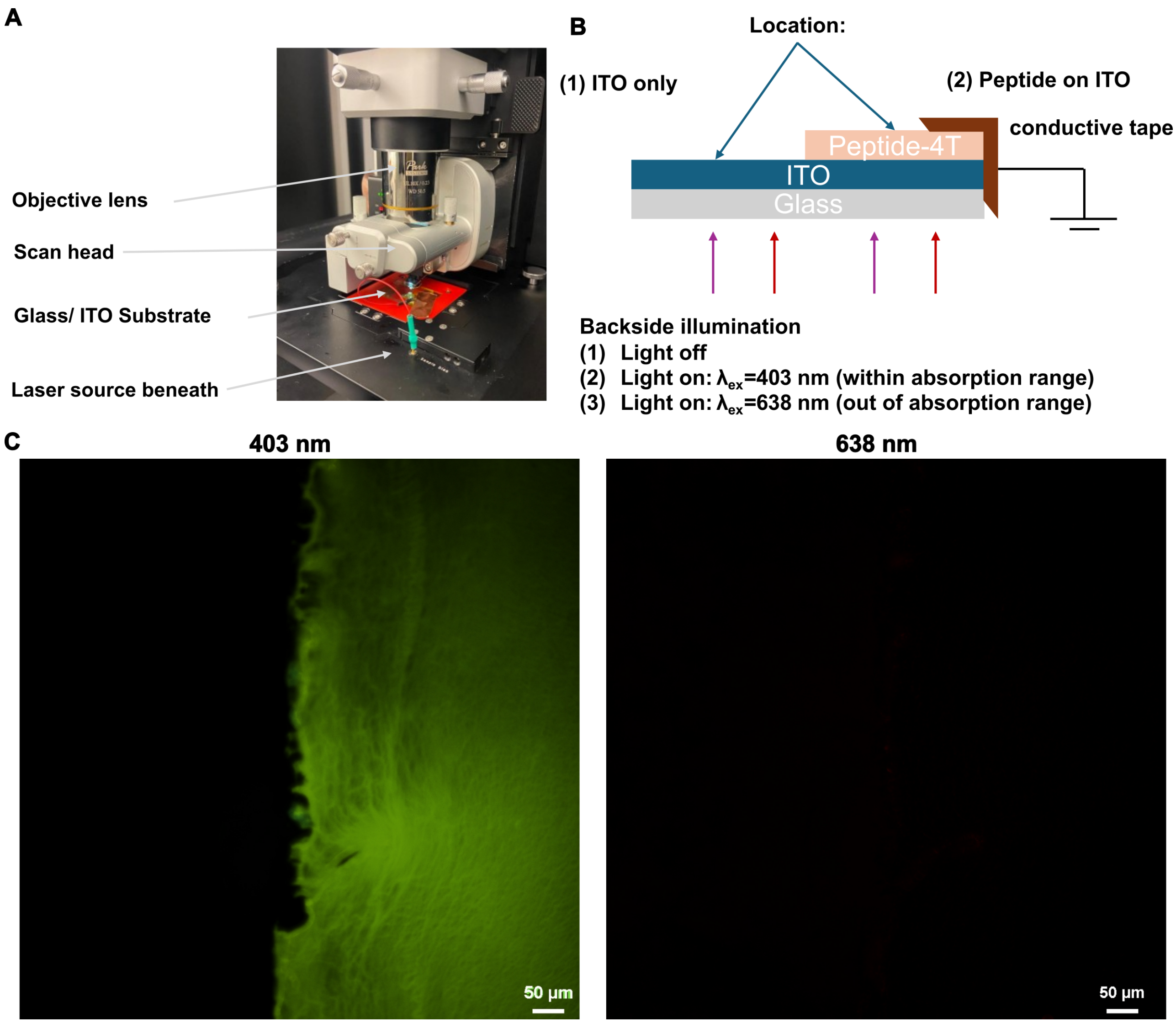

Figure S6. KPFM and AFM setup for DGREFEFK$_a$-4T ITO substrates. (A) Image and (B) schematic of KPFM set up to test photoresponsivity. (C) Spectral confocal microscopy images of the ITO glass half-coated with Peptide-4T under 403 nm and 638 nm excitation.

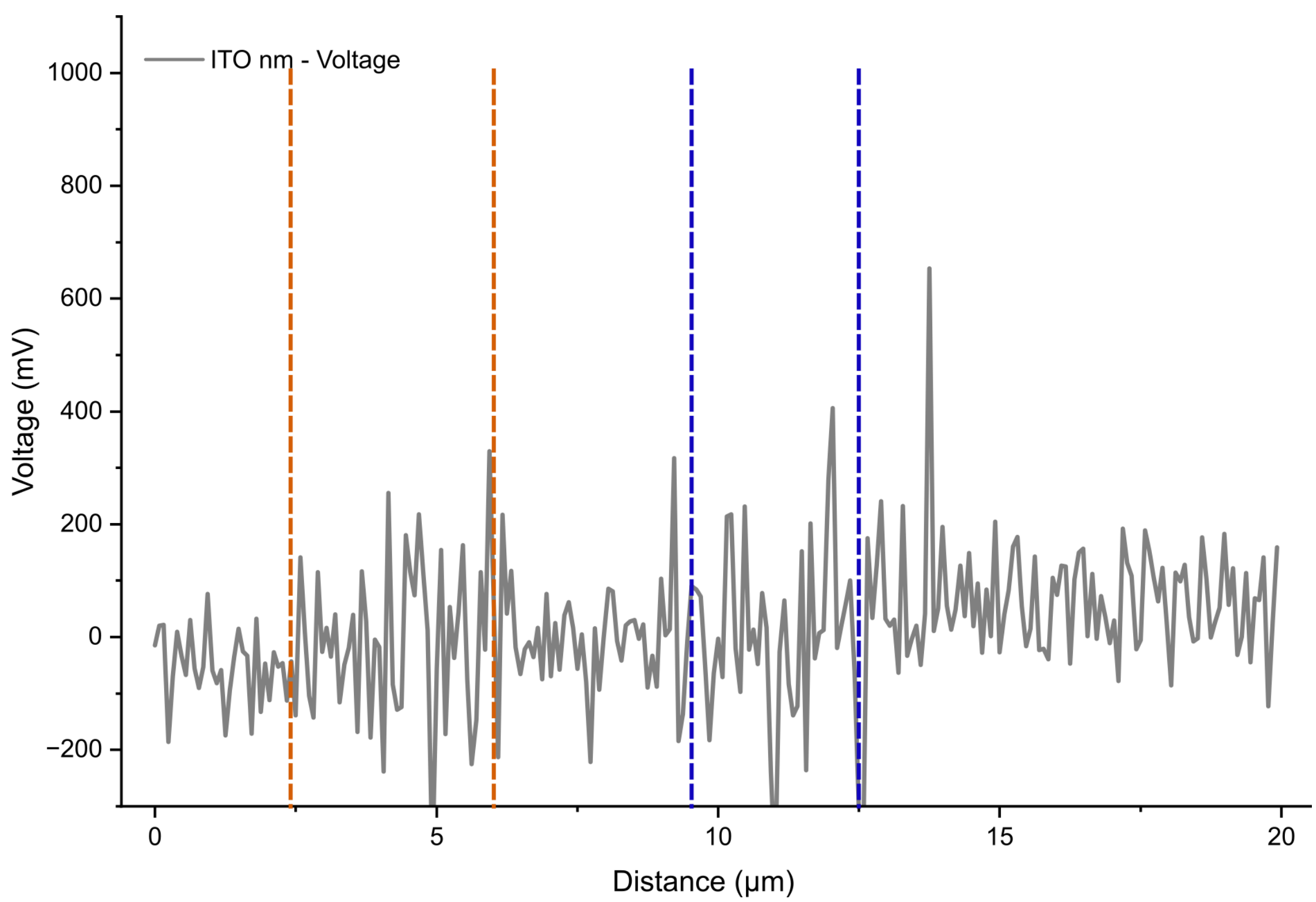

Figure S7. KPFM of ITO half of substrate without any peptide-4T. The 638 nm light was turned on during the duration between the dashed orange lines, while the 403 nm light was on in the period between the two blue dashed lines. There is no visible change in the surface potential in response to either wavelength of light.

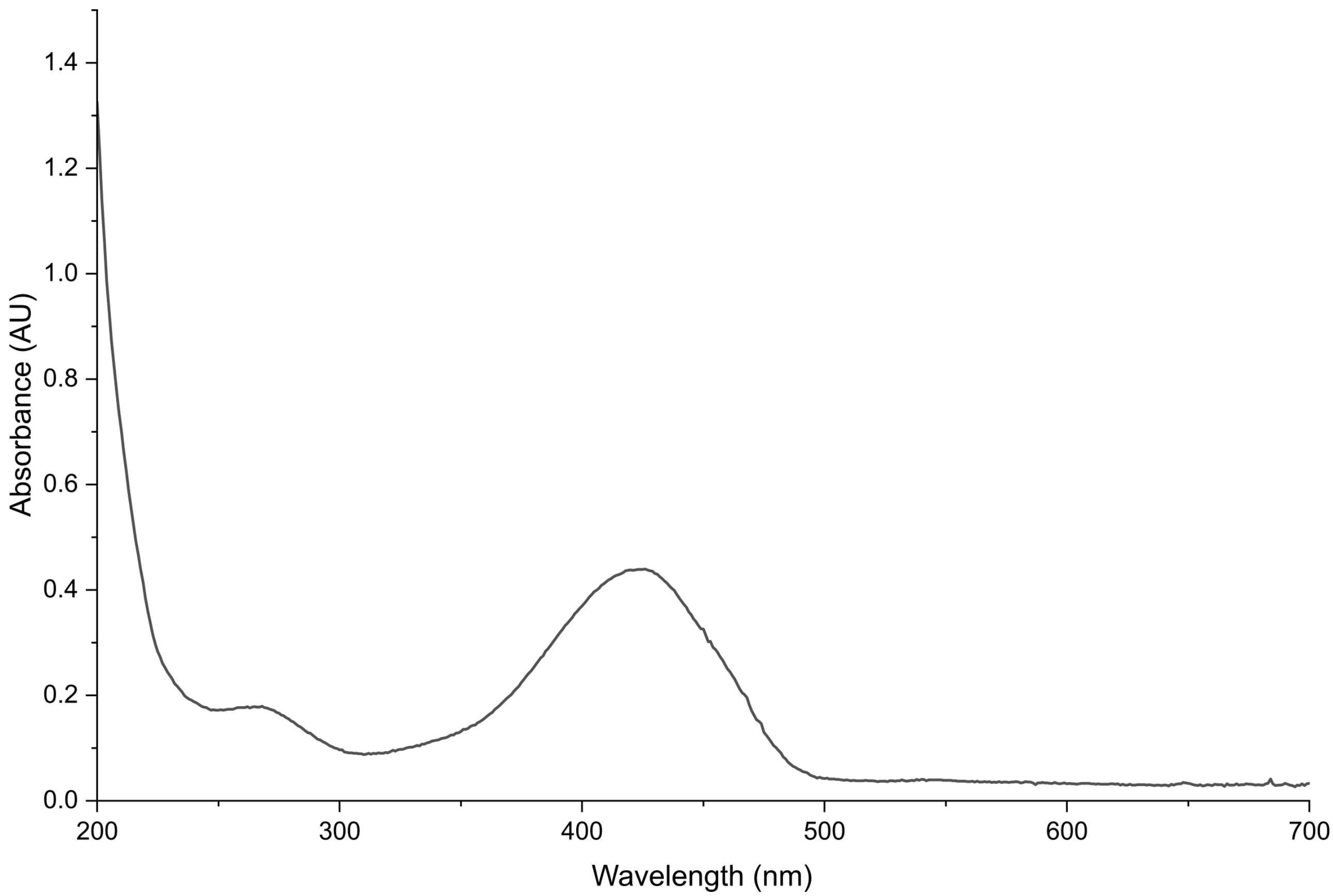

Figure S8. Absorbance spectrum of 0.1 mM DGREFEFK$_a$-4T in UltraPure water at neutral pH.

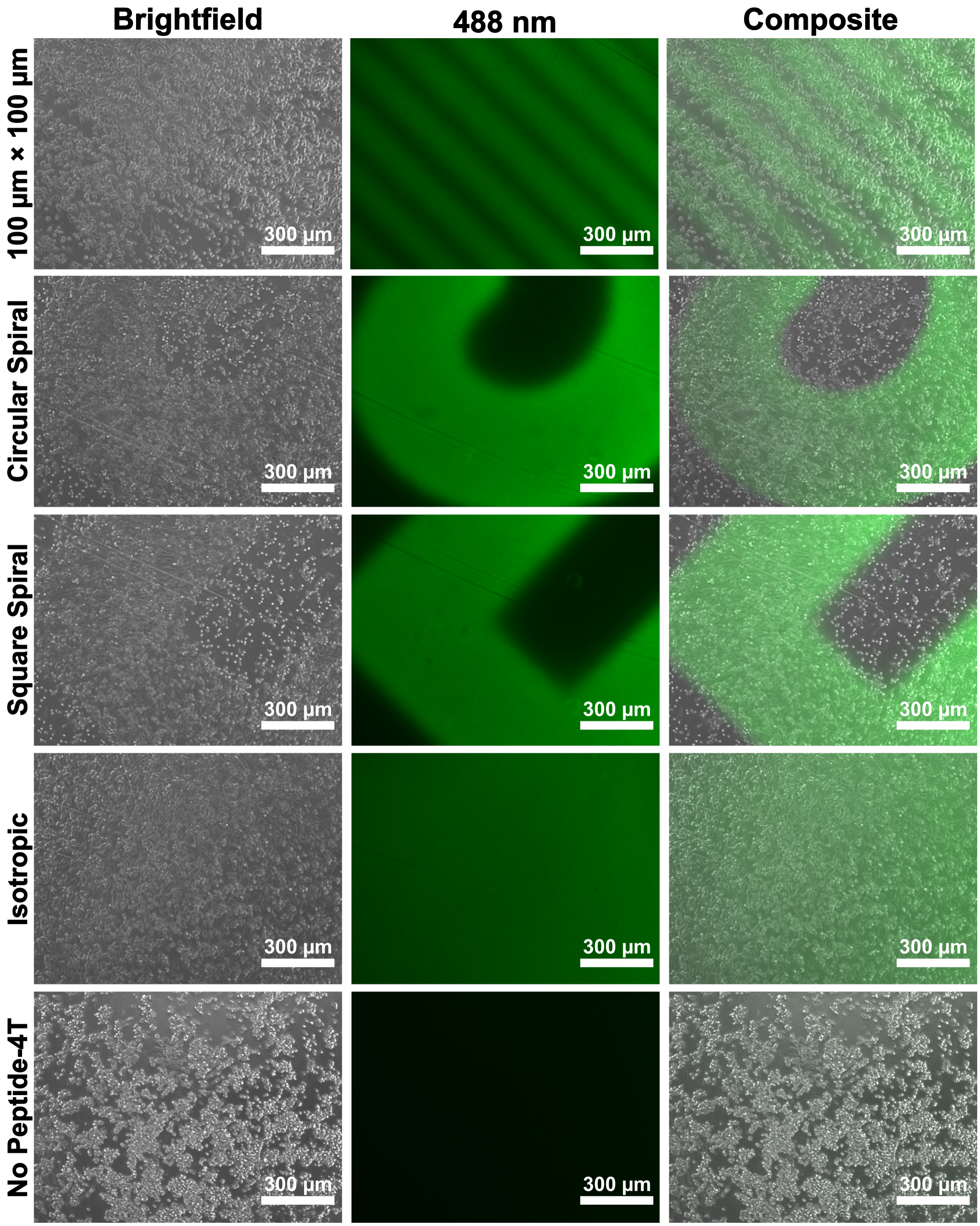

Figure S9. Brightfield images of NRVMs 24 hours after seeding and 488 nm fluorescent images of the DGREFEFK$_a$-4T patterns at each region of interest.

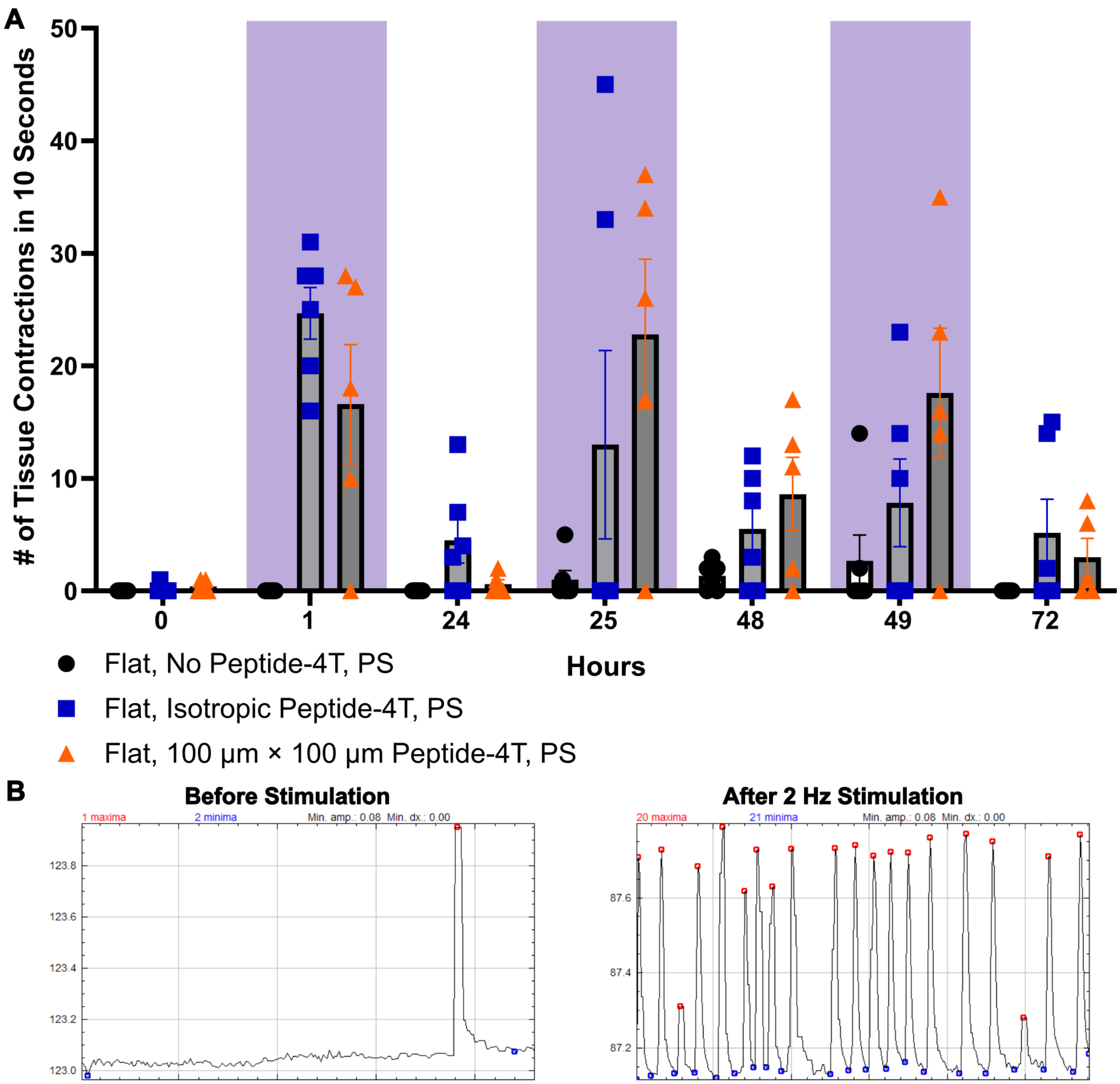

Figure S10. NRVM synchronous beating on Days 2-5 of cell culture in response to 415 nm photostimulation. (A) Number of cardiac contractions (beats) were tallied over 10 second videos to calculate the overall frequency. Data points collected immediately following a 415 nm stimulation are highlighted with a violet background N = 5-6 constructs per condition. Error bars = s.e.m. (B) Representative kymographs showing NRVM contractions before and after the 2 Hz 415 nm photostimulation on an isotropically patterned peptide-4T GelMA construct.

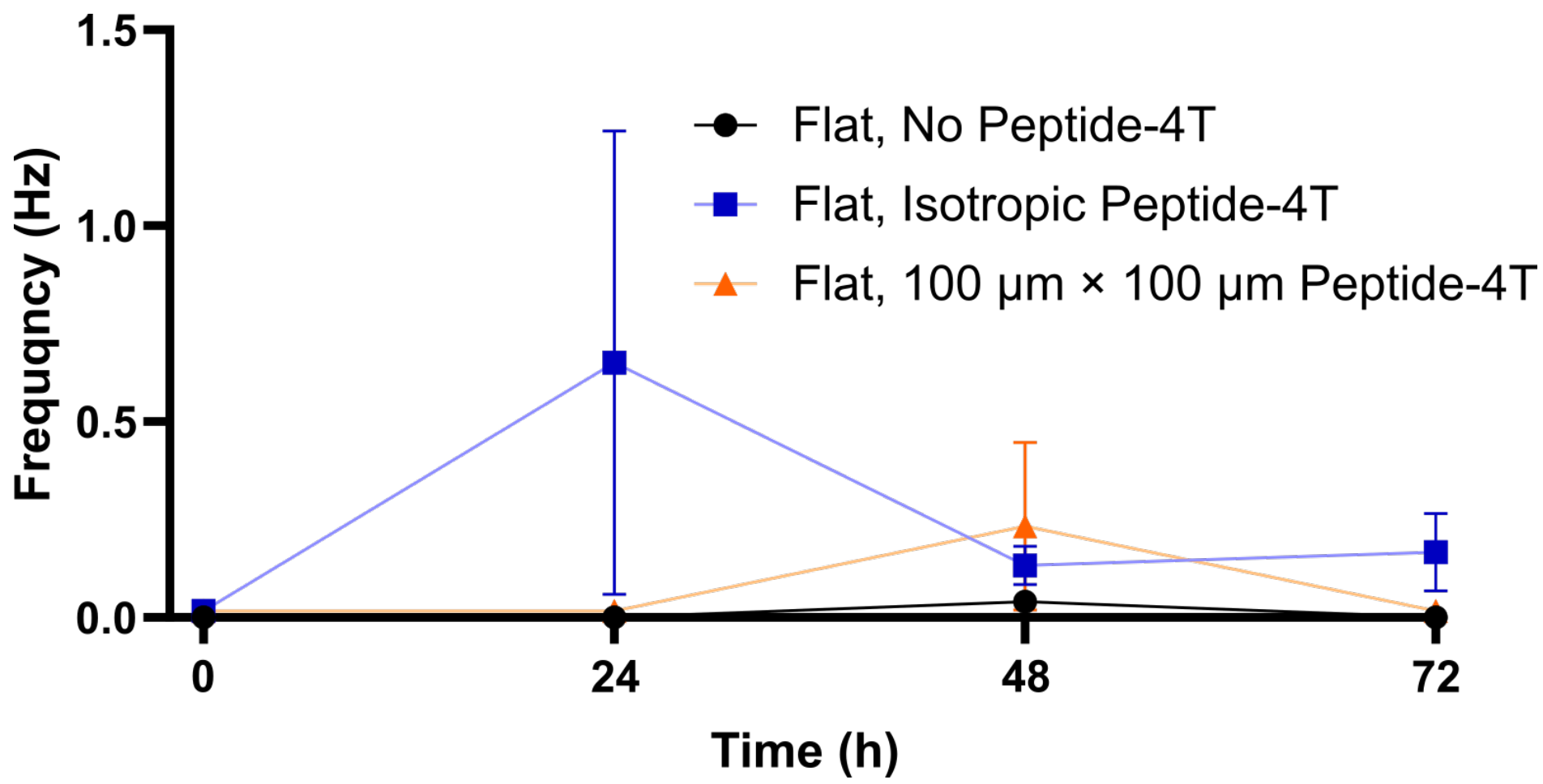

Figure S11. NRVM synchronous beating frequencies on Days 2-5 of cell culture, without any photostimulation. N =5-6 constructs per condition. Error bars = s.e.m.

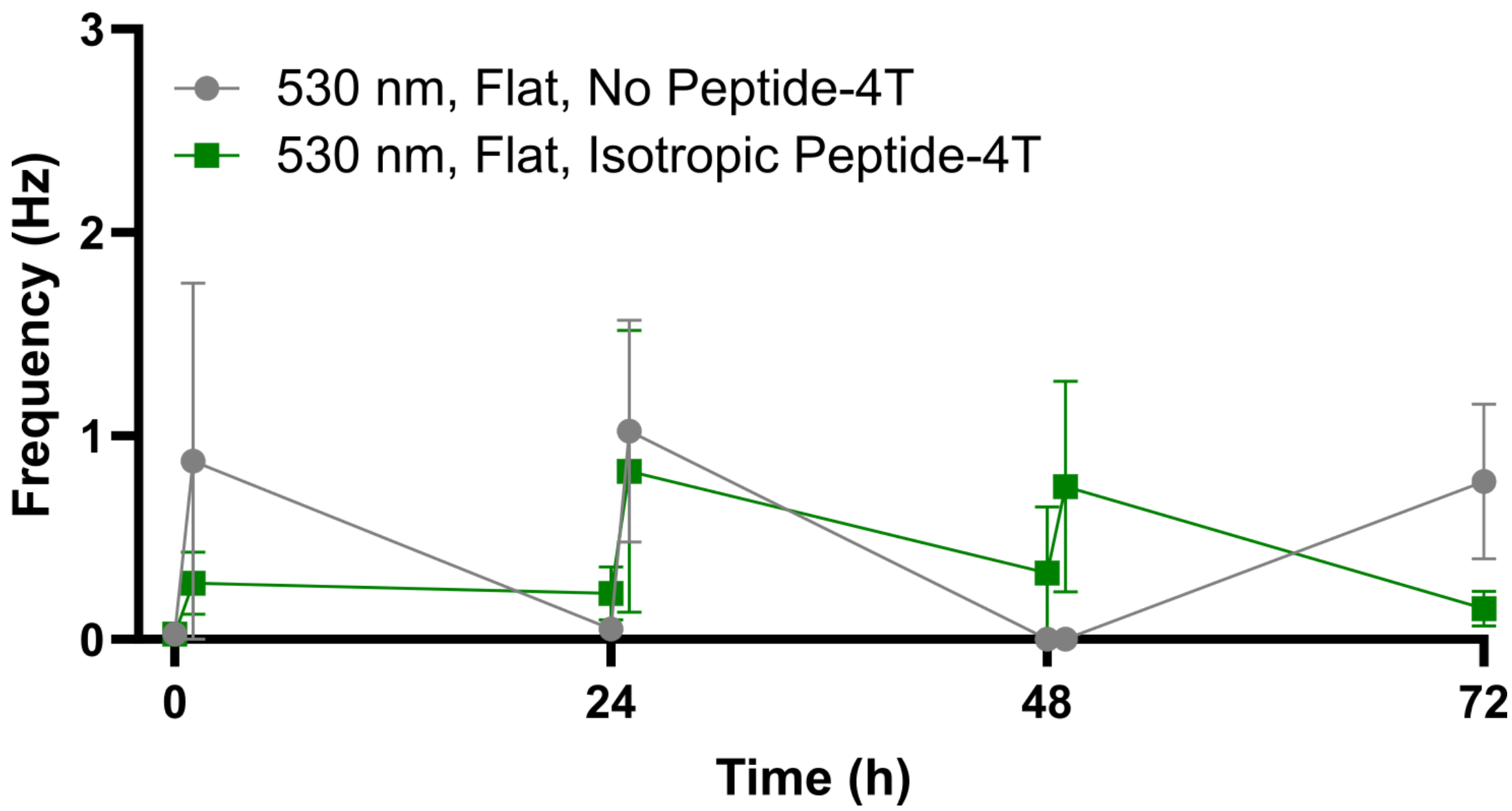

Figure S12. NRVM synchronous beating frequencies on Days 2-5 of cell culture, in response to 530 nm photostimulation. N = 4 constructs per condition. Error bars = s.e.m.

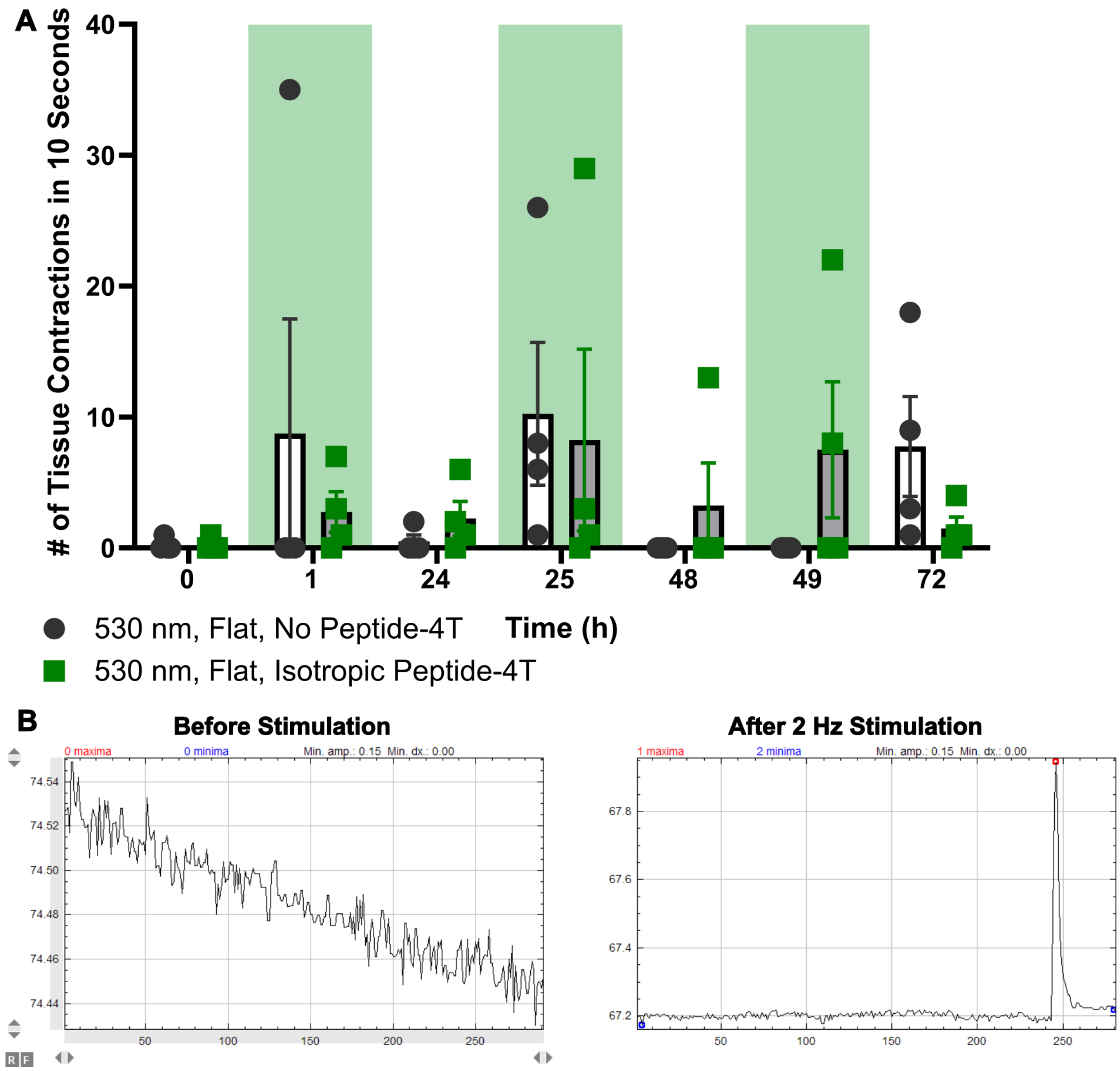

Figure S13. NRVM synchronous beating on Days 2-5 of cell culture in response to 530 nm photostimulation. Number of cardiac contractions (beats) were tallied over 10 second videos to calculate the overall frequency. Data points collected immediately following a 530 nm stimulation are highlighted with a green background. N = 4 constructs per condition. Error bars = s.e.m. Representative kymographs showing NRVM contractions before and after the 2 Hz 530 nm photostimulation on an isotropically patterned peptide-4T GelMA construct.

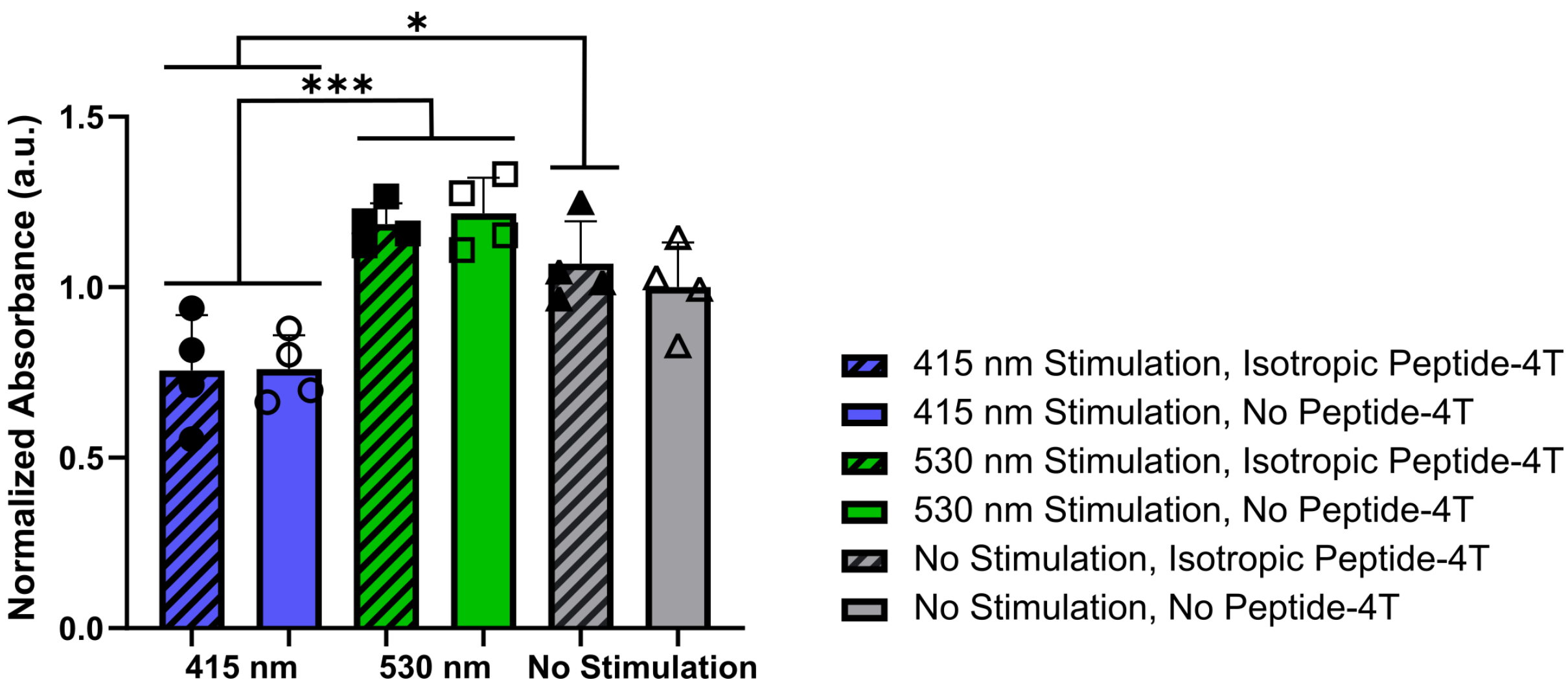

Figure S14. Cell viability in response to photostimulation via MTS colorimetric assay. There is a statistically significant decrease in absorbance for cells that underwent the 415 nm stimulation compared to the 530 nm stimulation and the isotropic peptide-4T coated GelMA constructs that did not undergo any stimulation. Results were normalized using the GelMA without peptide-4T and without any light stimulation. N = 4. Statistical significance calculated via one-way ANOVA and Tukey's HSD. *$p < 0.05$, ***$p < 0.001$. Error bars = s.e.m.

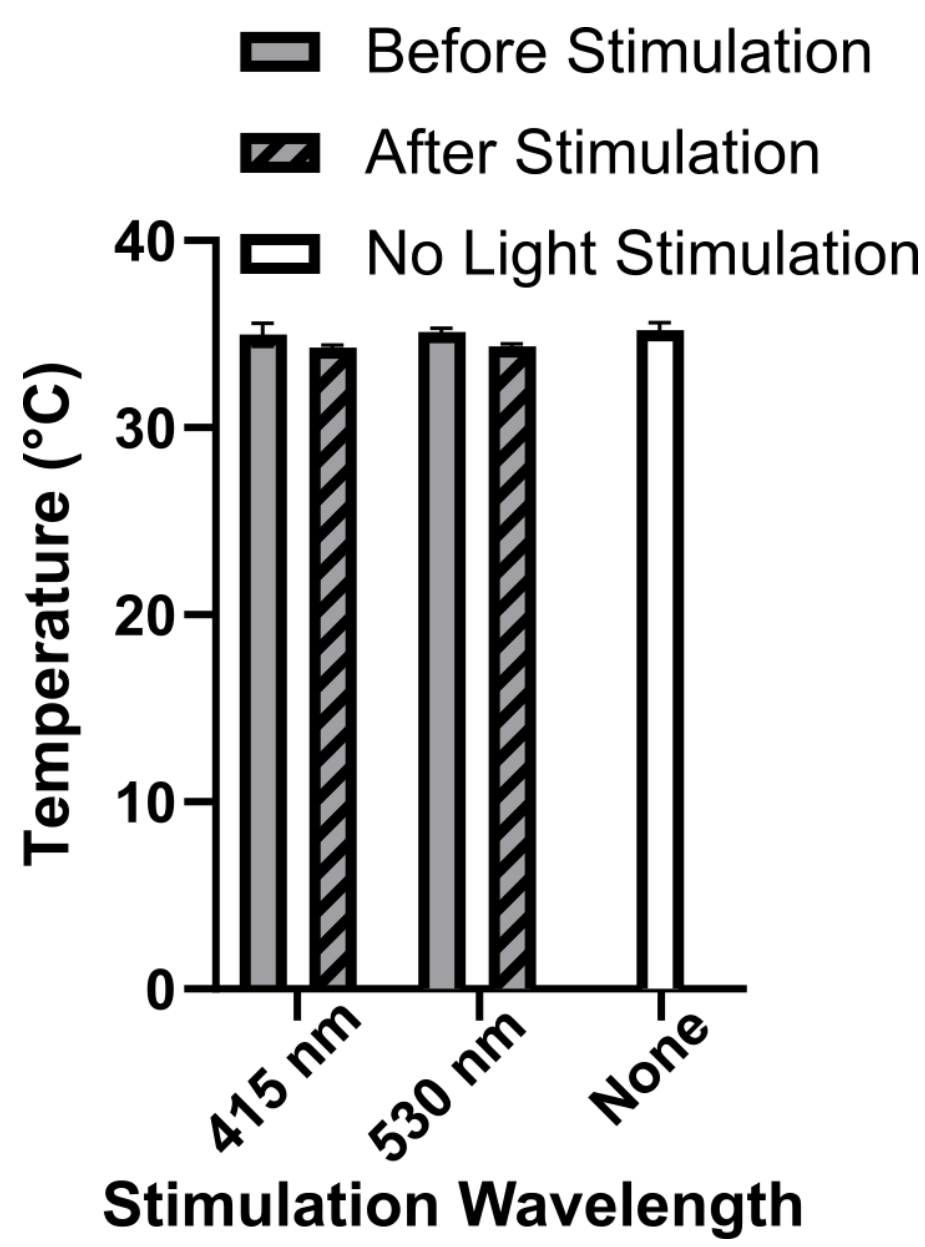

Figure S15. Temperature in the well of NRVMs on isotropically coated peptide-4T GelMA, as measured with an infrared thermometer before and after photostimulation, as well as on a non-stimulated control samples. N = 4 per stimulation condition. Error bars = s.e.m.

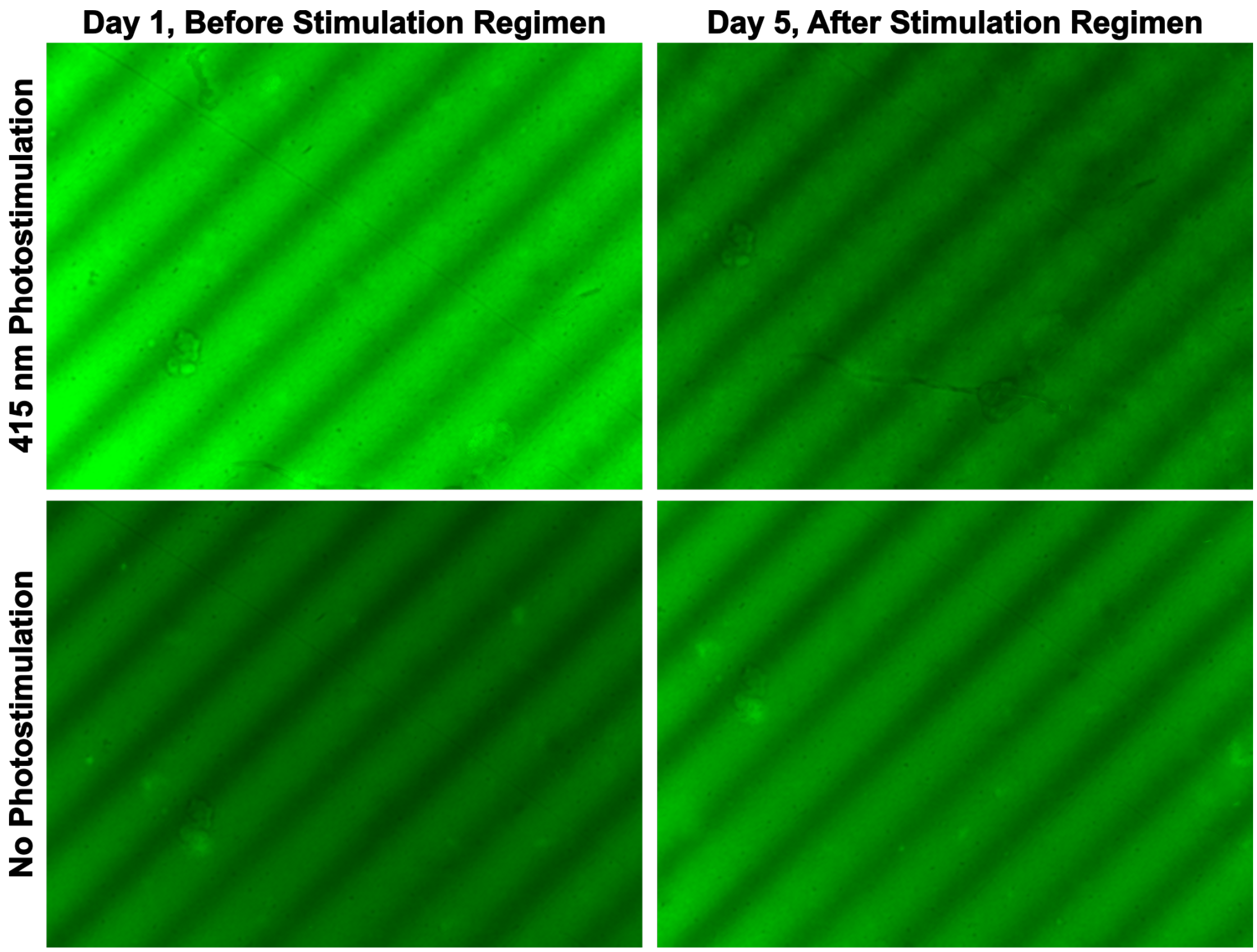

Figure S16. Pattern stability of 100 µm × 100 µm DGREFEFK$_a$-4T pattern in five days of a culture with NRVMs. The peptide-4T pattern is still present on samples that underwent the photostimulation regimen on Days 2, 3, and 4 of culture.

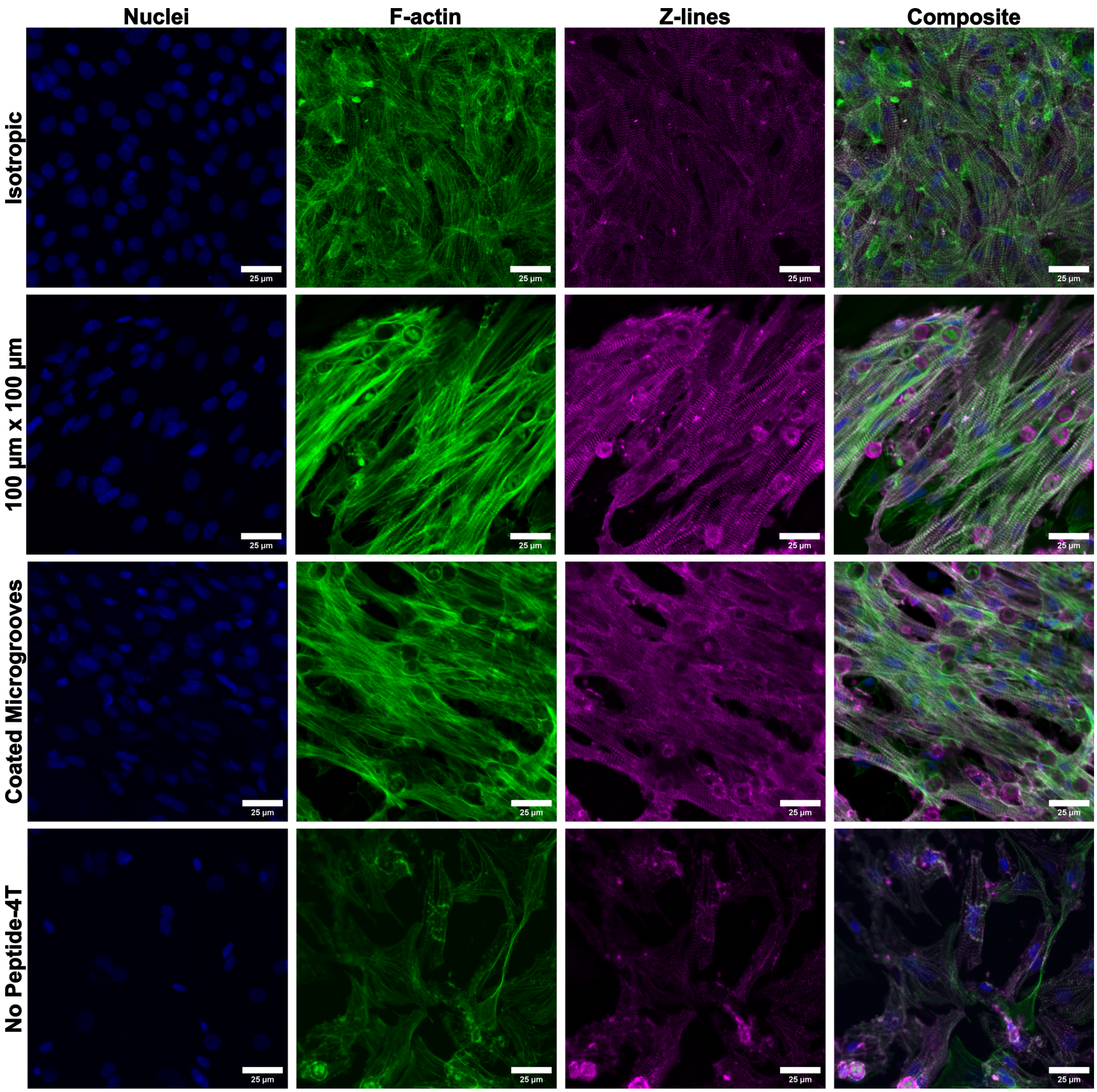

Figure S17. Representative images of immunostaining of NRVMs seeded on patterned peptide-4T constructs with a photostimulation regimen to investigate Z-line organization and architectural changes on various patterns. Nuclei, F-actin, and Z-lines are indicated in blue, green, and magenta respectively.

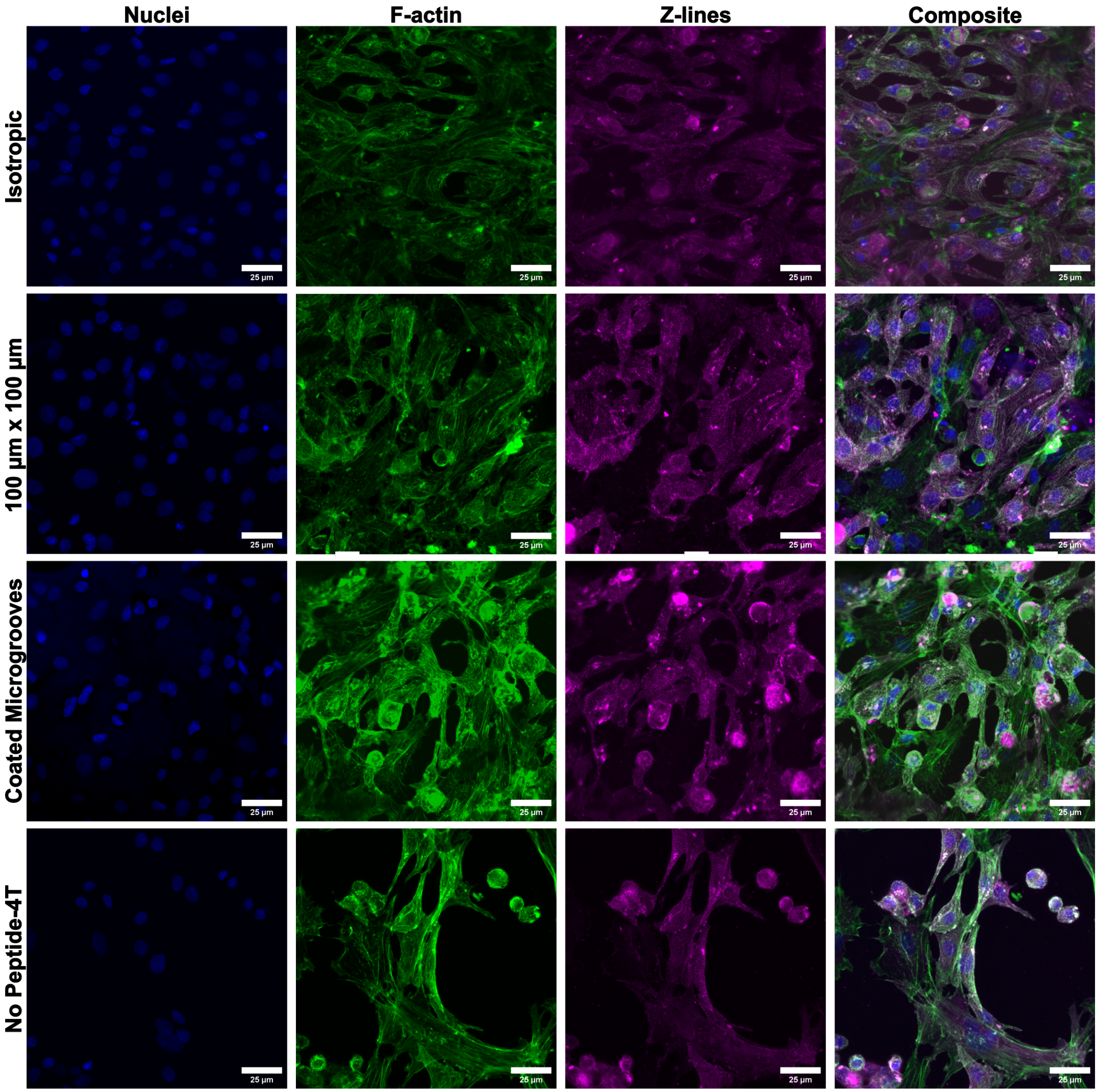

Figure S18. Representative images of immunostaining of NRVMs seeded on patterned peptide-4T constructs without a photostimulation regimen to investigate Z-line organization and architectural changes on various patterns. Nuclei, F-actin, and Z-lines are indicated in blue, green, and magenta respectively.

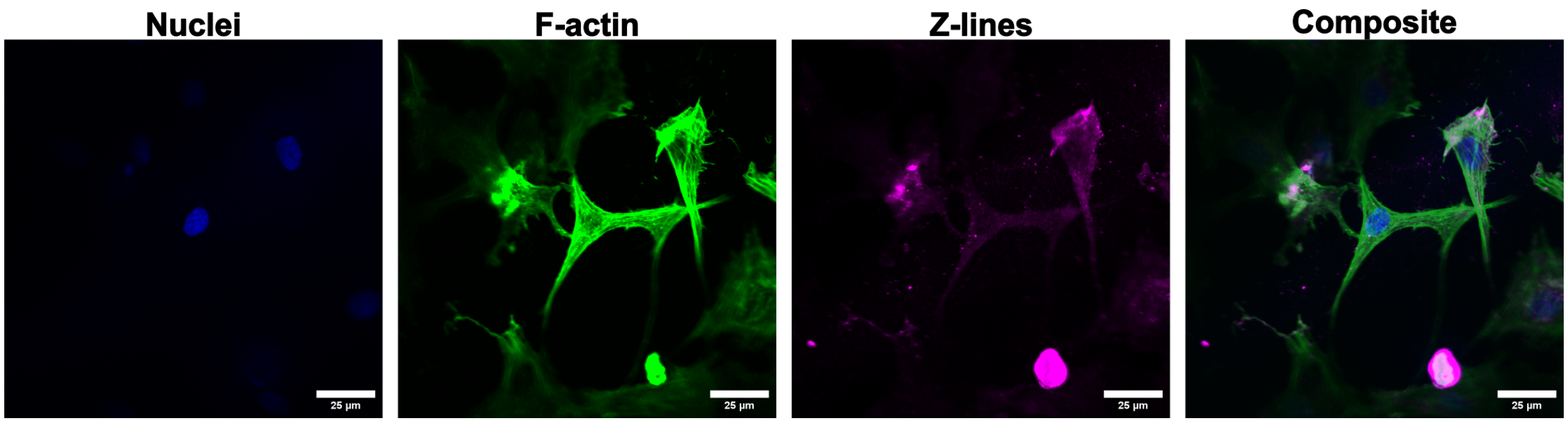

Figure S19. Representative images of immunostaining of NRVMs seeded on fibronectin coated, microgrooved constructs without peptide-4T to investigate Z-line organization and architectural changes. Nuclei, F-actin, and Z-lines are indicated in blue, green, and magenta respectively.

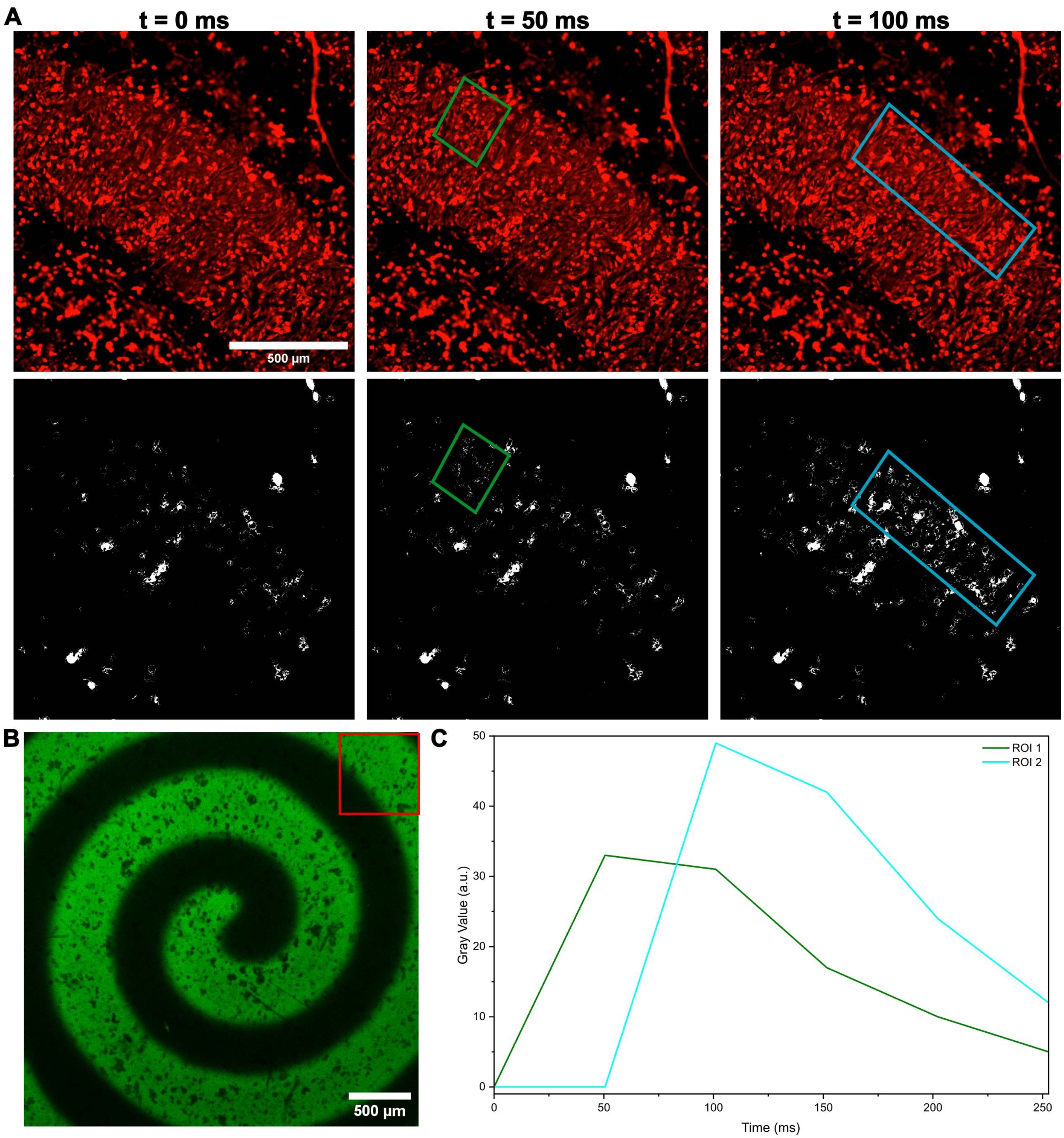

Figure S20. Visualized Ca^{2+} flux on a circular spiral peptide-4T patterned construct. (A) Fluorescence images of the Rhod-X-1 dye (top) and the processed and filtered image to remove outliers and help with analysis (bottom) at three different points in the calcium cycle. ROIs are boxed to indicate points where the calcium is starting to increase along the pattern. (B) Representation of where the images from A are being taken along the spiral pattern, as indicated by the red box. (C) Adjusted median gray values at the two ROIs (green and cyan) from (A) showing the progressive movement of calcium along the pattern.

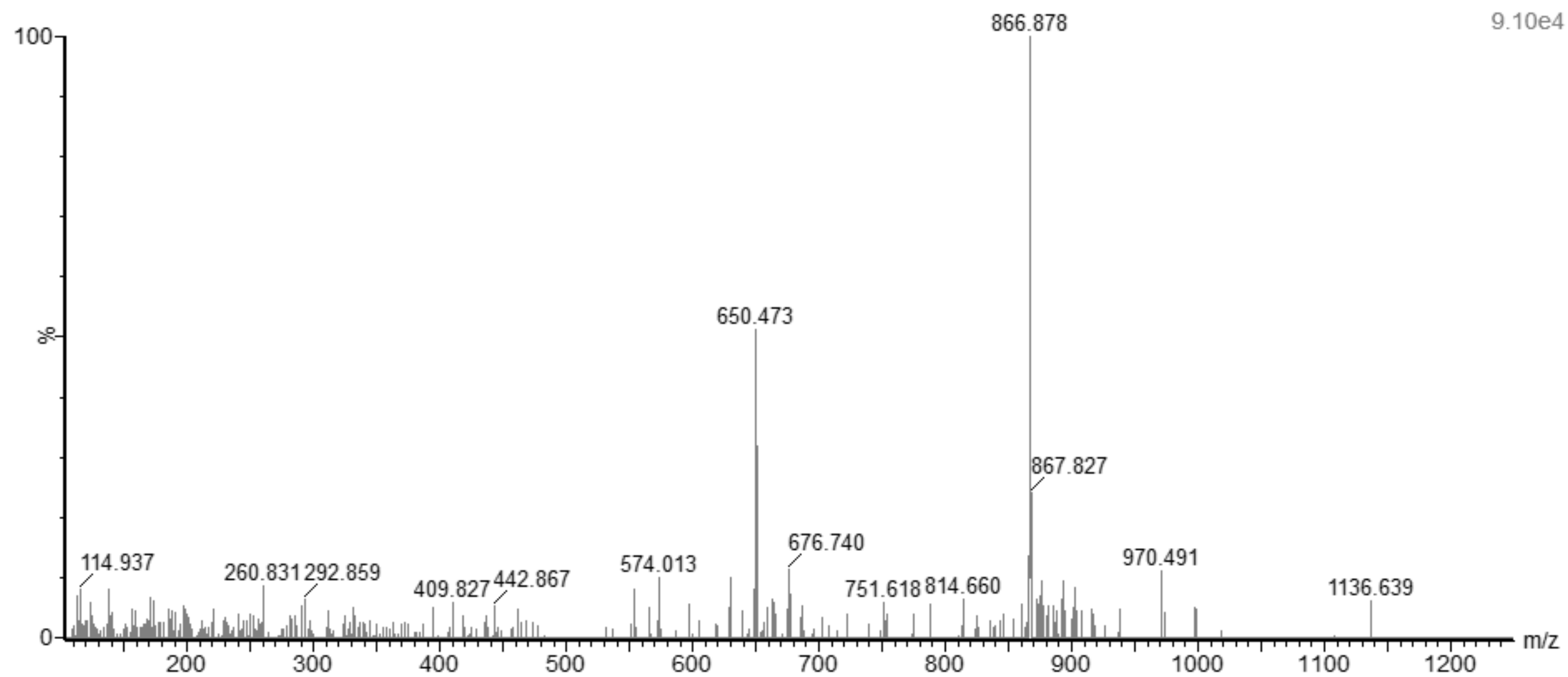

Figure S21. ESI-MS spectrum of DGREFEFKa-4T. m/z found $[M\text{-}3H]^{3-}/3$: 866.88, $[M\text{-}4H]^{4-}/4$: 650.47; calculated $[M\text{-}3H]^{3-}/3$: 866.97, $[M\text{-}4H]^{4-}/4$: 649.98. Final yield (by mass) for this batch of DGREFEFK_a-4T is 9.51%, calculated from an actual yield of 62 mg (0.024 mmol) and a theoretical yield of 652 mg (0.25 mmol).

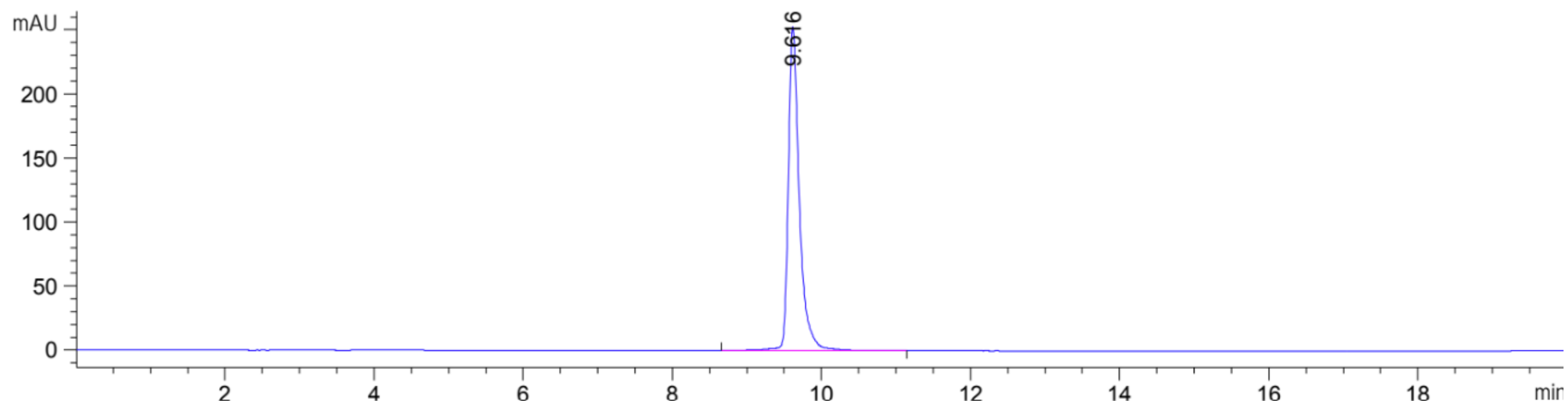

Figure S22. Purity characterization for DGREFEFK$_a$-4T samples via analytical HPLC (recorded at 400 nm). Analytical HPLC integration indicates a 100% purity of the DGREFEFK$_a$-4T sample. Final yield for this batch of DGREFEFK$_a$-4T is 9.51% calculated from an actual yield of 62 mg (0.024 mmol) and a theoretical yield of 652 mg (0.25 mmol).

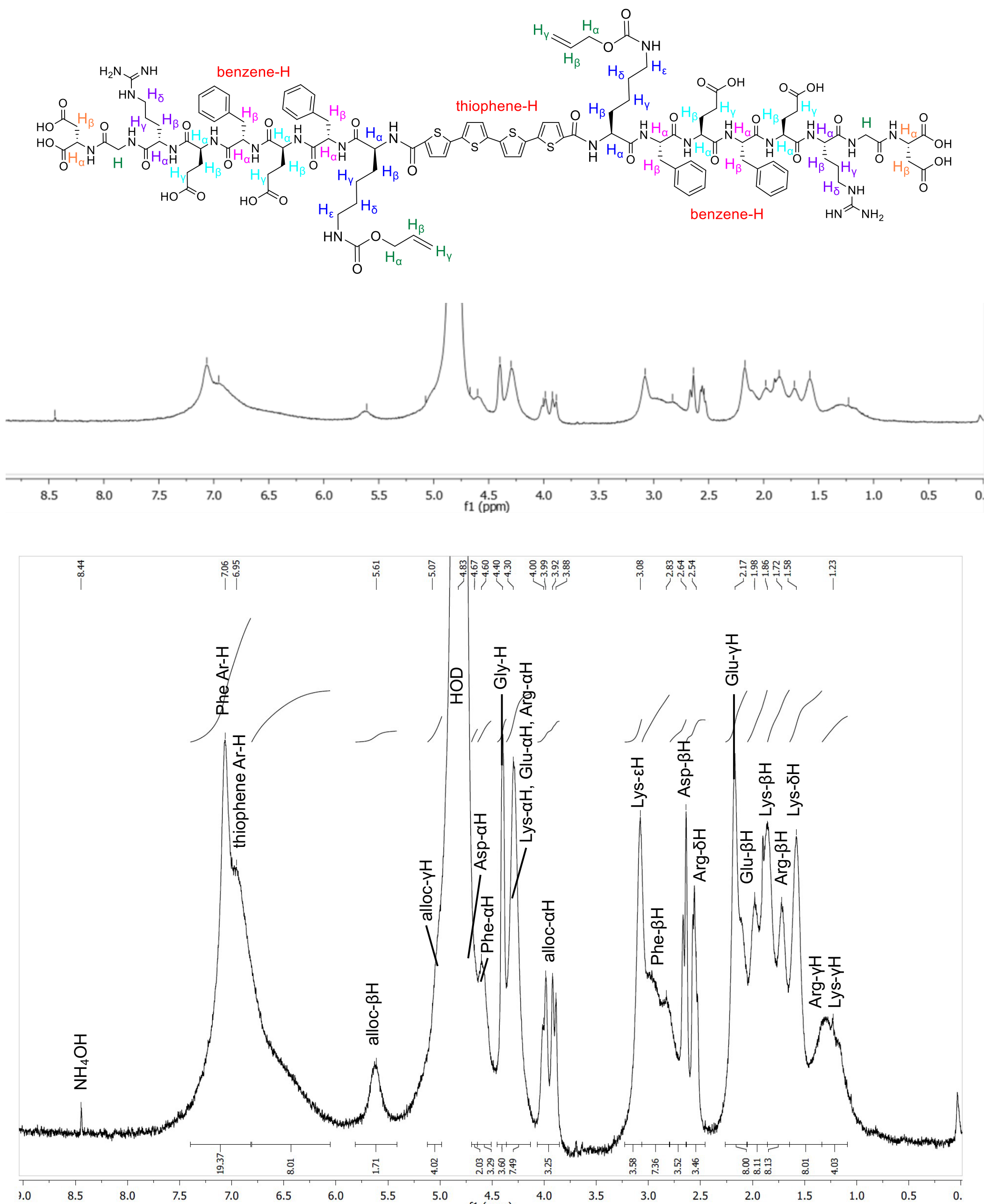

Figure S23. ^{1}H NMR spectrum of **DGREFEFKa-4T** (500 MHz, NaOD in D_2O, 10 mM). Peak assignments shown in the bottom spectrum; δ, ppm: 8.44 (ammonium formate), 6.82-7.39 (br, benzene Ar-H, 20H), 6.06-6.81(br, thiophene Ar-H, 8H), 5.41-5.81 (br, alloc-βH, 2H), 4.98-5.12 (br d, alloc-γH, 4H), 4.83 (HOD), 4.64-4.70 (br, Asp-αH, 2H), 4.64-4.51 (br, Phe-αH, 4H), 4.37-4.45 (s, Gly-H, 4H), 4.13-4.37 (br m, Lys_{alloc}-αH, 2H; Glu-αH, 4H; Arg-αH, 2H), 3.85-4.06 (d, alloc-αH, 4H, J= 15 Hz), 3.06-3.20 (br m, Lys_{alloc}-εH, 4H), 2.80-3.06 (br, Phe-βH, 8H), 2.64-2.80 (d, Asp-βH, 4H, J= 15 Hz), 2.48-2.64 (t, Arg-δH, 4H, J= 5 Hz), 2.05-2.26 (br, Glu-γH, 8H), 1.86-2.05 (br, Glu-βH, 8H), 1.64-1.86 (br, Lys_{alloc}-βH, 4H; Arg-βH, 4H), 1.33-1.64 (br m, Lys_{alloc}-δH, 4H; Arg-γH, 4H), 1.09-1.33 (br m, Lys_{alloc}-γH, 4H).

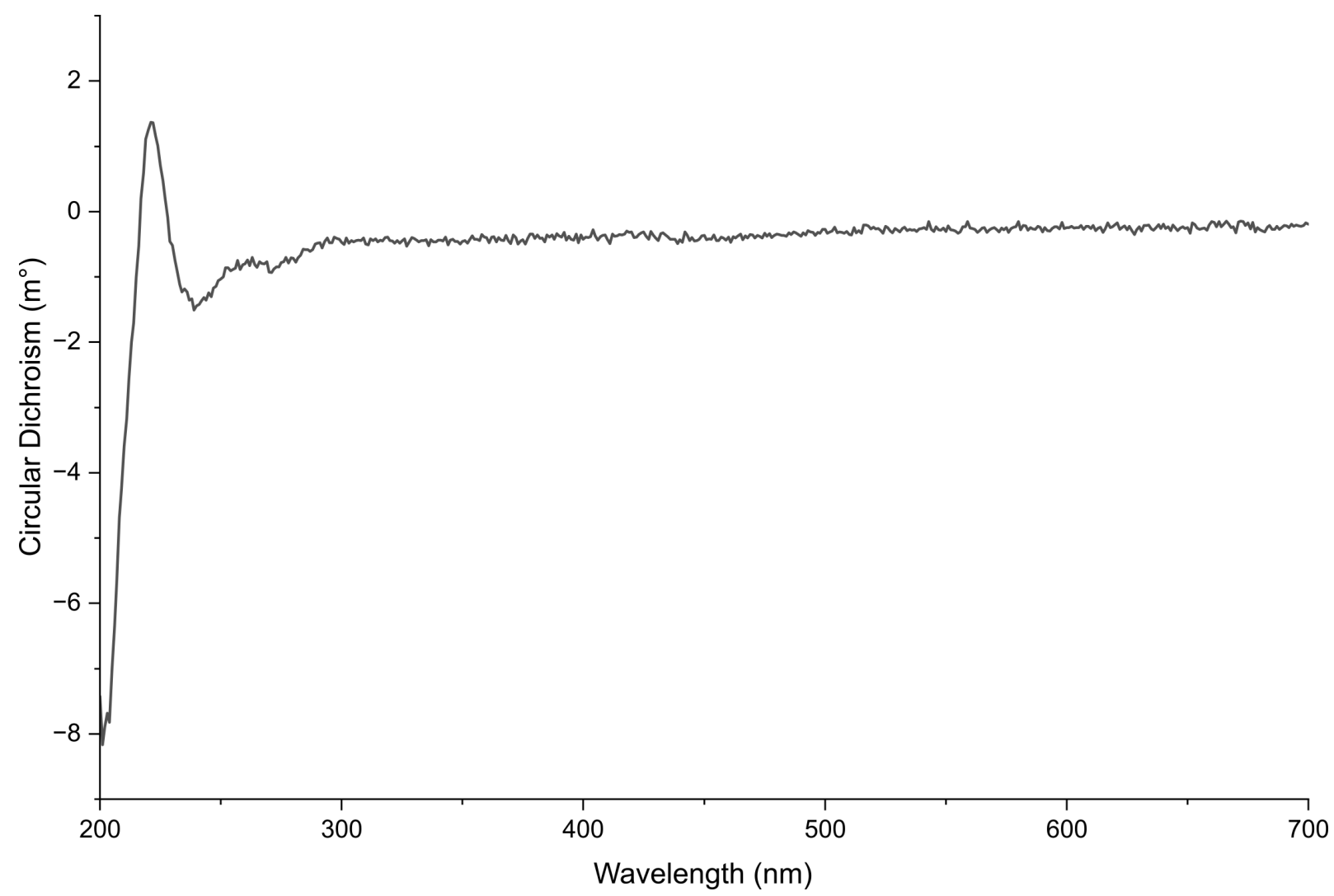

Figure S24. Circular dichroism (CD) spectrum of DGREFEFK$_a$-4T as 0.1 mM solution in UltraPure water at neutral pH.

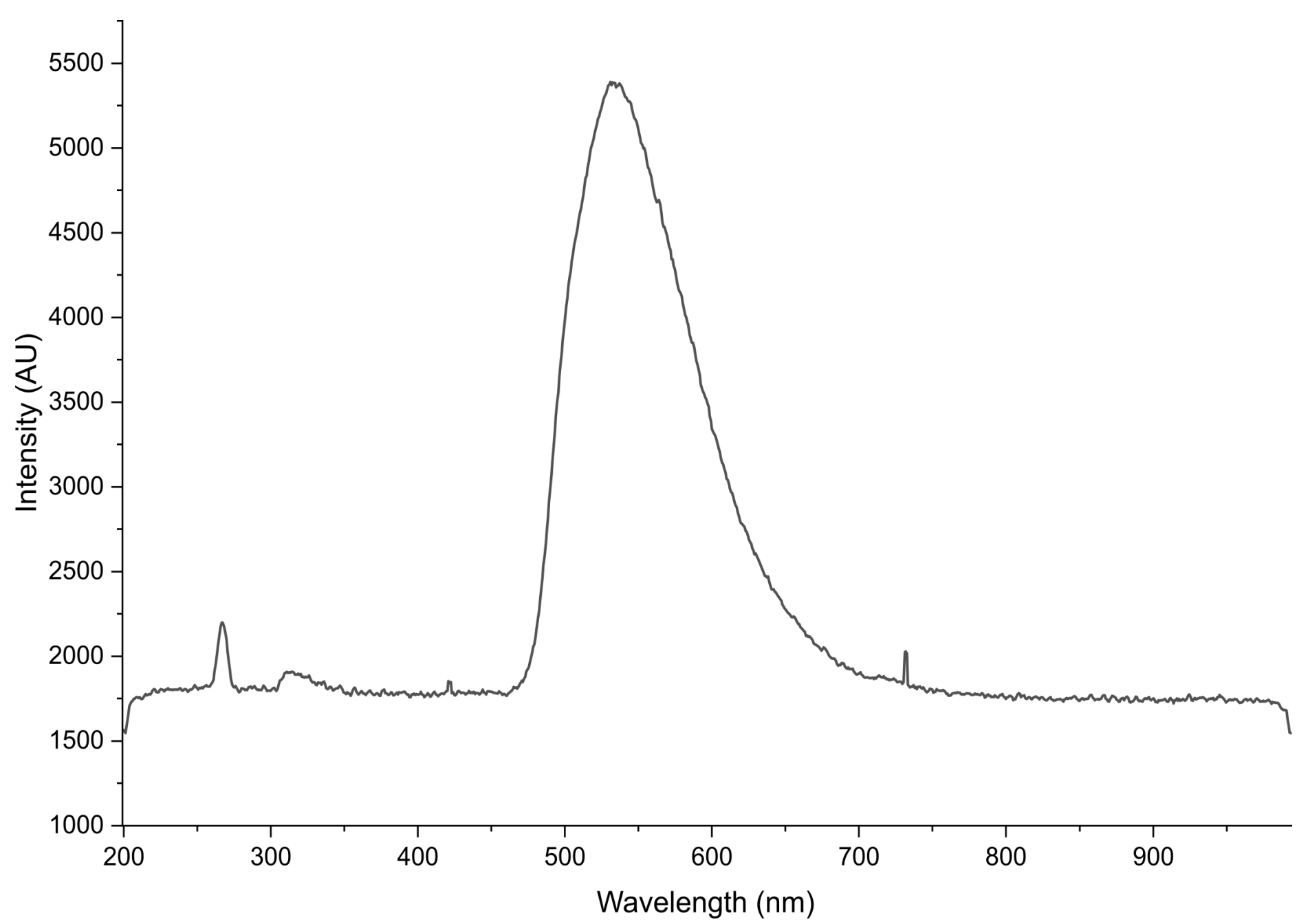

Figure S25. Photoluminescence (PL) spectrum of DGREFEFK$_a$-4T; maximum emission peak intensity around 550 nm. The PL data was collected from a sample of a 0.1 mM solution of DGREFEFK$_a$-4T in UltraPure water at neutral pH.
